# Accuracy and technical characteristics of CYP2C19 point of care tests: a systematic review

**DOI:** 10.1080/14622416.2024.2392479

**Published:** 2024-09-04

**Authors:** Eve Tomlinson, Chris Cooper, Hayley E Jones, Catalina Lopez Manzano, Rachel Palmer, Joe Carroll, Ayman Sadek, Nicky J Welton, Mariska Leeflang, Penny Whiting

**Affiliations:** a Population Health Sciences, Bristol Medical School, University of Bristol, Bristol, UK; b South West NHS Genomic Medicine Service Alliance; c Amsterdam University Medical Centers, University of Amsterdam

**Keywords:** antiplatelet therapy, clopidogrel, *CYP2C19* testing, diagnostic test accuracy, meta-analysis, systematic review

## Abstract

**Aim:** To assess the accuracy and technical characteristics of *CYP2C19* point of care tests (POCTs).

**Patients & methods:** Systematic review of primary studies, in any population or setting, that evaluated POCTs for detecting *CYP2C19* loss of function (LOF) alleles.

**Results:** Eleven studies provided accuracy data (eight Spartan; one Genomadix Cube; one GMEX; one Genedrive). The POCTs had very high sensitivity and specificity for the alleles they tested for. Twenty-two studies reported technical characteristics: POCTs were easy to operate and provided results quickly. Limited data were reported for test failure rate and cost.

**Conclusion:** *CYP2C19* POCTs may be a useful alternative to laboratory-based testing to guide antiplatelet therapy. Further data are required on accuracy (GMEX; Genedrive), test failure and cost (all POCT).

## Abstract (plain language summary)

Some people have loss of function (LOF) alleles in the *CYP2C19* gene, which reduce the effectiveness of clopidogrel (antiplatelet medicine). Point of care tests (POCT) can be used near-patient to quickly detect these alleles and guide alternative treatment. In this systematic review, we assessed the accuracy and technical characteristics of *CYP2C19* POCTs for the detection of *CYP2C19* LOF alleles.

Systematic review of primary studies, in any population or setting, that evaluated POCTs for detecting *CYP2C19* loss of function (LOF) alleles.

Eleven studies provided accuracy data: eight for Spartan versions of the Genomadix Cube *CYP2C19* system (detects LOF alleles: *2,*3); one for the current Genomadix Cube *CYP2C19* system (*2,*3), one the GMEX system (*2,*3); one the Genedrive *CYP2C19* ID Kit (*2,*3,*4,*8,*35). The POCTs had very high sensitivity and specificity for the alleles they tested for – Genomadix: 100% summary sensitivity (95% CI: 0.93–1.00) and specificity (95% CI: 0.99–1.00); Genedrive 100% sensitivity (0.96–1.00) and specificity (0.98–1.00); GMEX 100% sensitivity (0.98–1.00) and specificity (0.98–1.00). Twenty-two studies reported on technical characteristics (16 Spartan RX, one Genomadix Cube, two Genomadix test (version unclear), one GMEX, two Genedrive). POCTs were easy to operate and provided results quickly (∼60–90 min). Limited data were reported for test failure rate and cost.

*CYP2C19* POCTs may provide a useful alternative to laboratory-based testing to guide antiplatelet therapy. Further accuracy data are required for the GMEX and Genedrive POCTs, and more test failure and cost data are needed for all POCTs.

## 1. Introduction

Clopidogrel is an antiplatelet medicine prescribed to prevent blood clots and secondary vascular events in adults with conditions such as recent myocardial infarction, recent ischemic stroke, transient ischemic attack, peripheral arterial disease, atrial fibrillation and acute coronary syndrome [1,2]. Clopidogrel works by inhibiting adenosine diphosphate-induced platelet aggregation [3]. However, before achieving its pharmacological effect, the drug needs to be metabolized into an active metabolite in the body. This process is patient-specific, and a substantial proportion of the population are less able to perform it effectively, known as “clopidogrel resistance” [3]. Clopidogrel resistance has been found to be associated with poor clinical outcomes in patients, including a higher risk of adverse secondary vascular events [4–7]. Patients who have clopidogrel resistance could benefit from using alternative antiplatelet therapies, such as ticagrelor, prasugrel, or dipyridamole and aspirin [8–12].

Clopidogrel resistance is often caused by variants in the *CYP2C19* gene, which encodes a protein needed to metabolize clopidogrel [2,13]. The *CYP2C19* gene is polymorphic and the Pharmacogene Variation Consortium has specified 35 allele haplotypes [2]. *CYP2C19* alleles are identified using star (*) nomenclature. Alleles are ordered into functional categories: loss of function (LOF; e.g. *2, *3, *8, *4, *35), decreased function (e.g. *9), normal function (e.g. *1) and increased function (*17) [2]. The combination of *CYP2C19* alleles predicts an individual's ability to metabolize clopidogrel. Generally, people with two normal function alleles (e.g. *1/*1) are categorized as “normal metabolizers”, people with one normal function and one LOF allele (e.g. *1/*2), or one LOF allele and one increased function allele (e.g. *2/*17) are “intermediate metabolizers” and people with two LOF alleles (e.g. *2/*3) are “poor metabolizers”. “Rapid metabolizers” have one normal and one increased function allele (e.g. *1/*17) and “ultra-rapid metabolizers” have two increased function alleles (e.g. *17/*17). Limited data are available for decreased function alleles, but people with one decreased function allele (e.g. *9) and one normal or increased function allele are classified as “likely intermediate metabolizers”, while people with one decreased function allele and one LOF allele are “likely poor metabolizers”.

The prevalence of *CYP2C19* alleles varies according to ethnicity. For example, in a large analysis of *CYP2C19* alleles in the US, the frequency of the LOF allele *2 was found to be 28.4% in East Asian, 31.8% in South Asian and 27.6% in Native Hawaiian and other Pacific Islander populations [14]. Whereas, its prevalence ranged from 11.9% in Middle Eastern populations to 17.5% in African American populations. The *3 LOF allele was found to be less common overall; it was reported to be most prevalent in Native Hawaiian and other Pacific Islander (6.5%) and East Asian (6%) populations. The *17 increased-function allele was most common in people of European (21.7%), Ashkenazi Jewish (21.4%), or Middle Eastern descent (21.7%).

Tests can be used to identify *CYP2C19* LOF alleles and to guide alternative antiplatelet treatment. Where used, *CYP2C19* tests have traditionally been conducted by genetic technologists within the laboratory, for example Sanger sequencing, next-generation sequencing and targeted gene variant detection. Such laboratory-based tests are able to target all LOF alleles and have the flexibility to change the alleles screened for as new evidence emerges. However, they require trained genetic technologists to conduct them and the time to results is often too long to be used to guide initial treatment [15]. To overcome these barriers, *CYP2C19* point of care tests (POCT) have been developed that can be conducted by healthcare professionals outside of a conventional laboratory setting [16]. POCTs only target specific *CYP2C19* LOF alleles, but they have the potential to provide results more quickly, to inform initial treatment decisions. Several POCTs are licensed to be used for the detection of *CYP2C19* LOF alleles: the Genomadix Cube *CYP2C19* system (detects *2,*3,*17 alleles), the GMEX system (detects *2,*3,*17 alleles) and the Genedrive *CYP2C19* ID Kit (detects *2,*3,*4,*8,*17,*35 alleles). These POCTs analyze DNA obtained from a buccal sample to detect *CYP2C19* alleles. Further test details are summarized in Supplementary Material Section 1.

POCTs are increasingly being used within most healthcare settings [17], including in critical care (e.g. international normalized ratio POCT for anticoagulation monitoring [18]), primary care (e.g. C-reactive protein POCT to identify patients at high risk of respiratory infections, inflammatory or cardiovascular diseases [19]), at-home (pregnancy tests; personal glucose meters for glucose monitoring; blood sugar strips for diabetes [20]), and in low resource settings with limited laboratory access (e.g. POCT for HIV, syphilis and malaria [20]). Despite POCT being commonly used for different conditions, challenges in implementation exist, including training needs for non-laboratory staff who use POCT, integration of POCT into the clinical pathway and cost [21].

This systematic review explored the diagnostic accuracy of POCTs for detecting the presence of specific *CYP2C19* LOF alleles, and the technical characteristics (e.g. ease of use, test failure rate) of *CYP2C19* POCTs.

## 2. Methods

This review aligns with published guidance on systematic review conduct [22–24], and is reported as per PRISMA-DTA (Supplementary Material Section 5) [25,26]. The review protocol is listed on Open Science Framework [27].

### 2.1. Eligibility criteria

Primary studies in any population or setting that evaluated the Genomadix Cube *CYP2C19* system (including previous versions of this test by the same manufacturer which are deemed to be largely equivalent to the Cube: Spartan RX *CYP2C19* system and Spartan FRX *CYP2C19* system), the Genedrive *CYP2C19* ID Kit, or the GMEX system, for the detection of *CYP2C19* LOF alleles, were eligible for inclusion. These POCTs were selected as they are marketed to be used for the detection of *CYP2C19* LOF alleles (see Supplementary Material Section 1).

We identified two additional POCTs that had been evaluated for *CYP2C19* LOF allele detection: the Verigene system (Luminex) and the Q3 system (ST Microelectronics). These were not included in the review because the test developers confirmed that the Verigene system is no longer used for *CYP2C19* and that the Q3 system is used for research purposes only.

For the accuracy evaluation, studies were eligible for inclusion if they used a laboratory-based reference standard and if they reported data on sensitivity and specificity (or sufficient information to construct 2 × 2 data). Studies were not eligible for inclusion if they took samples from patients and tested them multiple times, without reporting results at the sample level. For evaluation of technical characteristics, studies were eligible for inclusion if they provided data on one or more of the following outcomes: test failure rate, time to results, ease of use of test, cost (any costs reported by a study e.g. device cost, staff time/training).

### 2.2. Search strategy

The bibliographic search was structured as follows: (((terms for point of care tests) AND (terms for *CYP2C19* OR terms for Clopidogrel)) OR (known NCT numbers)) and it is reported in Supplementary Material Section 2 with a search narrative [28]. We searched the following databases from inception to May 2023, with an update search in June 2024, without limit on publication type, date or language of publication: MEDLINE (MEDALL; via OVID), Embase (via Ovid), CINAHL (via EBSCO Host); Cochrane's CENTRAL database (via Wiley). We also searched ClinicalTrials.gov, WHO ICTRP, Scan Medicine (https://scanmedicine.com/) and the Retraction Watch Database (http://retractiondatabase.org/RetractionSearch.aspx).

To identify any further potential studies to include, we checked the websites of the manufacturers of the POCTs in scope, reviewed company submissions provided as part of a NICE diagnostic accuracy review [7] and undertook a forward citation search on the primary study report for all studies included at full-text in Science Citation Index Expanded (via Clarivate). Lastly, we checked the reference lists of included studies.

### 2.3. Study selection

Two reviewers (ET; CC) screened titles and abstracts of reports identified by the searches, independently. Full texts of reports considered potentially relevant were retrieved and assessed for inclusion by two reviewers (ET; CC). Any disagreements were resolved by discussion.

### 2.4. Data extraction

One reviewer (ET) extracted data into piloted forms in Microsoft Word. Data were checked by a second reviewer (CC/PW). As per the protocol, the following data were extracted: funding, start date, study location, study design, inclusion and exclusion criteria, participants (condition, age, ethnicity, sex), POCT details, reference standard test details and data on the following outcomes: test accuracy, test failure rate, time to results, ease of use of test and cost [27].

Accuracy data were extracted as pre-specified in the protocol [27]. As noted, each person has two *CYP2C19* alleles; some are associated with normal function (e.g. *1), some with LOF (e.g. *3) and some with over-metabolism (e.g. *17). Treatment for over-metabolizers does not differ to the other categories, so in this review these alleles were grouped with normal function alleles. Individuals could therefore belong to one of three groups: normal function (e.g. *1/*1; *1/*17), one LOF allele (e.g. *2/*1; *3/*1; *3/17; *2/*17), two LOF alleles (e.g. *2/*2; *3/*3; *3/*2). We dichotomized these groups into two categories: alleles for normal function and alleles for no function. A “positive” test result was defined as the presence of at least one LOF allele (non-functional). A positive reference standard was as reported by the study (detection of alleles identifiable by the evaluated POCT). The reference standard was also dichotomized so that a “poor metabolizer” was specified as having at least one LOF allele. Where multiple sets of 2 × 2 data were reported by a study (e.g. for different tests, thresholds, reference standards, or alleles), all data were extracted. When required, we contacted authors of studies to request information to generate 2 × 2 data [27].

### 2.5. Risk of bias assessment

One reviewer (ET) used the QUADAS-2 tool to assess the risk of bias in included diagnostic test accuracy (DTA) studies [29]. One signaling question about test thresholds and one question about the time interval between index test and reference standard were omitted from QUADAS-2 because genetic tests do not have a threshold in the usual test accuracy sense, and the test results are not affected by time. Additionally, due to the broad scope of this review, applicability was not considered within the tool, but possible sources of heterogeneity were considered in the synthesis. Risk of bias assessment was checked by a second reviewer (CC/PW) and any disagreements were resolved by discussion.

### 2.6. Data synthesis

Analyzes were stratified by POCT and outcome [27]. We narratively synthesized study details, risk of bias and results. Sensitivity and specificity for POCTs were calculated from 2 × 2 data, assuming that the laboratory reference standards had categorized all study participants correctly. Where multiple studies evaluated the accuracy of the same POCT, summary estimates of sensitivity and specificity with 95% confidence intervals (*CIs*) were calculated using bivariate random effects meta-analysis, assuming binomial likelihoods [30,31]. Coupled forest plots of sensitivity and specificity displayed results from individual studies and summary estimates, to allow for the visual assessment of heterogeneity. As there was homogeneity of estimates across studies, heterogeneity was not investigated formally. We did not identify sufficient data to conduct a meta-analysis for any of the technical characteristics outcomes.

### 2.7. Differences between protocol & review

We amended our inclusion criteria to note that studies were not eligible for inclusion if they took samples from patients and tested them multiple times, without reporting results at the sample level.

## 3. Results

### 3.1. Search results

**Figure 1.**
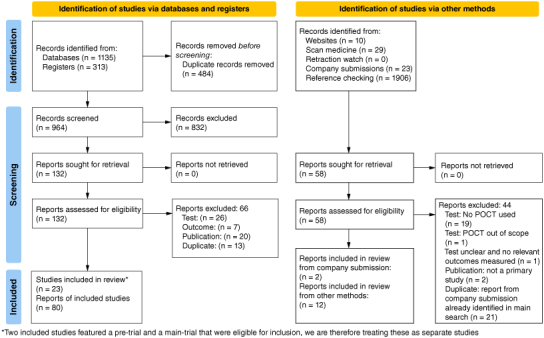
PRISMA chart showing study selection process.

Searches via databases and registers identified 964 unique reports. Additional methods of study identification identified 1968 reports. In total, 23 studies (80 reports) were included in the review. Eleven studies reported accuracy data, 22 studies reported technical characteristics data and 10 studies reported data on both types of outcome. The study selection process is outlined in Figure 1 , and a summary of included studies and studies excluded at full text screening is provided in Supplementary Material Section 3.

### 3.2. Accuracy of POCTs for detecting the presence of *CYP2C19* LOF alleles

Eleven studies (34 reports), provided data on the accuracy of *CYP2C19* POCTs [32–41]. Of these, one provided details of a pre-trial and a main-trial which both met inclusion criteria, and therefore were included as separate studies [32]. One study was reported as a trial registration only with results included in the trial registry entry [34], and one study was reported only in an “ID Kit and Performance Data brochure” provided by Genedrive [38]. All other studies were published as full journal articles and all studies were published in English.

One study evaluated the accuracy of the Genedrive *CYP2C19* ID Kit [38], one evaluated the GMEX system [39], one evaluated the Genomadix Cube [40] and the other eight studies evaluated Spartan versions of the Genomadix Cube *CYP2C19* system (seven evaluated Spartan RX and one evaluated Spartan FRX). The Spartan tests are generally considered to be equivalent to the Genomadix Cube *CYP2C19* system, so were evaluated along with the study on Genomadix Cube as a single group referred to as “Genomadix (Spartan) *CYP2C19* tests”, unless referring to a specific test. Table 1 provides a summary of the characteristics of the studies that provided test accuracy data, and full study details are reported in Supplementary Material Section 4.

**Table 1.** Characteristics of studies providing test accuracy data for *CYP2C19* POCT.

	Genomadix (Spartan) *CYP2C19* tests	Genedrive *CYP2C19* ID Kit	GMEX system
# studies	9	1	1
Reference	[32–37,40,41]	[38]	[39]
Version of test	**7** Spartan RX **1** Spartan FRX **1** Genomadix Cube	N/A	N/A
Population	**6** PCI **1** Healthy people **1** Cardiology/angiology/neurology patients **1** Not reported	**1** Donor specimens	**1** Healthy people/people with cardio- and cerebro-vascular diseases
Country	**3** Canada **2** International (Canada/US/Mexico/South Korea) **1** South Korea **1** Malta **1** Switzerland **1** Czech Republic	**1** Not reported	**1** China
Funding	**2** Industry – test manufacturer **3** Non-industry **1** Industry – other **1** Mixed (industry and non-industry) **1** Not funded **1** Not reported	**1** Industry – test manufacturer	**1** Non-industry
Alleles targeted	**6** 2*, 3* and 17* 2 2* 1 2*, 17*	**1** *2, *3, *4, *8, *35, *17	**1** 2*, 3* and 17*
Who administered POCT	**5** Not reported **1** Onsite testing staff **1** Staff physicians **1** Trial nurses **1** Clinical pharmacist researcher	**1** Not reported	**1** Doctors, nurses, clinical researchers
Reference standard	**3** Taqman **1** Direct DNA sequencing **1** Bidirectional sequencing **2** MassARRAY **1** Sanger sequencing **1** Taqman & GEN ID	**1** Agena MassARRAY and/or Taqman	**1** Sanger sequencing

Note: One report for the Genomadix (Spartan) *CYP2C19* test contains details pertaining to an eligible pre-trial and main-trial, which have been counted as separate studies in this table and review.

PCI: percutaneous coronary intervention.

The study of the Genedrive *CYP2C19* ID Kit was funded by the test manufacturer and the study of the GMEX System was funded by non-industry organizations. Of the studies evaluating Genomadix (Spartan) *CYP2C19* tests, three were funded by industry (two test manufacturer; one other industry organizations), three were non-industry funded, one was funded by both industry and non-industry, one did not report funder details and one (the study of Genomadix Cube) reported that it was not funded.

The study of the Genedrive *CYP2C19* ID Kit recruited donor specimens (no further information provided), and the GMEX system study recruited healthy people and people with cardiovascular and cerebrovascular diseases. Six studies of Genomadix (Spartan) *CYP2C19* tests recruited patients undergoing percutaneous coronary intervention (PCI), one recruited healthy people (pre-trial test validation), one (the study of Genomadix Cube) recruited patients from specialties of cardiology, angiology and neurology with indication for clopidogrel genotyping and one did not report population details (trial registration only). Overall, the 11 studies enrolled participants and tested one sample per person – the number of participants in the studies ranged from 35 to 2587 (mean: 420).

Three studies of Genomadix (Spartan) *CYP2C19* tests took place in hospitals in Europe, three were undertaken in the University of Ottawa Heart Institute in Canada, one in Dong-a University Hospital in South Korea, and two (reported in the same publication) were multi-national and conducted in clinical trial centers in USA/Canada/South Korea/Mexico. The study of the GMEX system was conducted in six hospitals in China, and the Genedrive accuracy study did not report on setting or country. Most studies did not report who administered the POCT, but in the five studies that did, POCTs were administered by clinical staff (e.g. nurses, doctors and clinical researchers).

Of the studies that evaluated Genomadix (Spartan) *CYP2C19* tests, six reported on *2, *3 and *17 alleles (all alleles that can be detected by the tests), two reported on *2 only and one reported on *2 and *17. The Genedrive study reported on all alleles that can be detected by the test (*2, *3, *4, *8, *35, *17), as did the GMEX system study (*2, *3, *17).

The reference standard was bidirectional sequencing in one study, direct DNA sequencing in one study, and Sanger sequencing in two studies. These tests can identify any LOF allele. Seven studies used targeted *CYP2C19* gene variant detection as the reference standard which has the ability to target all alleles but is often set up to target specific alleles: five of these used Taqman (two also used GEN ID and MassARRAY) and two studies used MassARRAY only. Supplementary Material Section 1 outlines the characteristics of these reference standard tests. In all studies, the laboratory tests only targeted the alleles that were targeted by the POCT. Due to this, accuracy estimates show the accuracy in identifying the alleles that the POCT can detect, rather than the accuracy of identifying any LOF variant.

All of the studies that provided accuracy data were judged to be at low risk of bias, except for the study of the *Genedrive CYP2C19 ID Kit*. This was rated as unclear risk of bias as it did not report information about the selection of the study population [38]. In the other studies, a variety of populations were enrolled and not always consecutively, but this was thought to be unlikely to affect estimates of POCT performance. Details about blinding of result interpreters were not always reported, but this was considered unlikely to have caused bias as the tests are objective in their interpretation. Supplementary Material Section 4 reports all risk of bias assessments.

**Figure 2.**
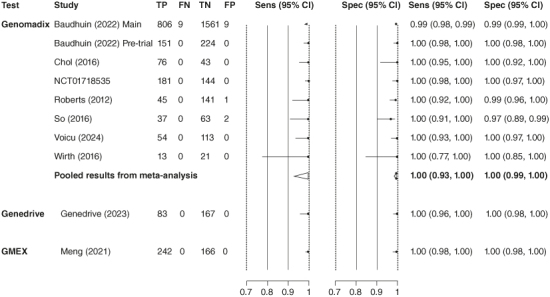
Paired forest plots of individual study estimates, stratified by test and summary estimates of sensitivity and specificity from meta-analysis, for studies that evaluated Genomadix (Spartan) *CYP2C19* tests.

Figure 2 presents paired forest plots of sensitivity and specificity (95% CI) estimates for the detection of LOF alleles, stratified by test. Summary estimates from meta-analysis are provided as diamonds on the plot for studies evaluating Genomadix (Spartan) *CYP2C19* tests (as these were evaluated by more than one study). Full results are reported in Supplementary Material Section 4.

### 3.3. Genomadix (Spartan) *CYP2C19* tests

Seven of the nine studies that provided accuracy data for Genomadix (Spartan) *CYP2C19* tests reported 100% sensitivity and specificity (this includes the study of Genomadix Cube [40], the most current version of the Genomadix test). The other two studies [32,35] did not report 2 × 2 data and the authors did not reply when we requested this information. One of these two studies reported “100% concordance” between the POCT and reference standard, but it did not report the number with and without LOF alleles, so we were unable to estimate 2 × 2 data [35]. For the other study, (Baudhuin 2022 main trial) we could estimate the data using details reported in the publication [32]. The paper reported sensitivity, specificity and the total number of people tested using Spartan RX (255/2641 did not have a Spartan RX result). The paper did not report the number of people tested with and without LOF alleles based on the reference standard for the ∼90% of samples that were tested with Spartan RX, but it provided the number of people with and without LOF alleles in the total sample. To estimate numbers with and without LOF in the tested sample, we made an assumption that the proportion of people with LOF alleles in the ∼90% subset was the same as the proportion in the total sample. We then applied sensitivity and specificity to these numbers to estimate the 2 × 2 table.

Summary sensitivity was 100% (95% CI: 93, 100). Summary specificity was also 100% (95% CI: 99, 100). There was no suggestion of any difference across the versions of the Genomadix test evaluated.

### 3.4. Genedrive *CYP2C19* ID Kit

The one study that provided accuracy data for the Genedrive *CYP2C19* ID Kit [38], judged to be at an unclear risk of bias, reported that it had 100% sensitivity (95% CI: 96, 100) and 100% specificity (95% CI: 98, 100) for the detection of *2, *3, *4, *8, *35 LOF alleles.

### 3.5. GMEX system

The one study providing accuracy data for the GMEX System, judged to be at low risk of bias, reported that it had 100% sensitivity (95% CI: 98, 100) and 100% specificity (95% CI: 98, 100) for the detection of *2 and *3 LOF alleles [39].

### 3.6. Discordant results

For Genomadix (Spartan) *CYP2C19* tests, the proportion of discordant results between the index test and the reference standard ranged from 0 to 2.9% and was <1% in six studies (Table 2). Although five studies reported discordant results, these only affected estimates of accuracy in three studies [32,36,41], as in the other two studies they did not affect the classification of the individual as a normal or poor metabolizer. The study that evaluated the Genedrive *CYP2C19* ID Kit reported discordant results for four samples, but these also did not affect the classification of the individual as a normal or poor metabolizer. There were no discordant results for the GMEX system.

**Table 2.** Overview of discordant results between *CYP2C19* POCT and laboratory reference standard tests.

Study	Test	Proportion discordant	Overview of discordant results	Impact on accuracy	Ref.
Baudhuin et al. (2022) – pre-trial	Spartan RX	2/373 (0.5%)	2 discordant initially due to pre-analytical sample mix-up at testing center. Samples concordant after being re-collected and re-tested.	None	[32,42]
Baudhuin et al. (2022) – main-trial	Spartan RX	21/2384 (0.9%)	21 discordant: 9 non-carrier on Spartan but *2 or *3 on TaqMan 9 heterozygous *2 or *3 on Spartan but non-carrier on TaqMan 1 heterozygous *2 on Spartan, but homozygous *2 on TaqMan	9 FN and 9 FP	[32,42]
Choi et al. (2016)	Spartan RX	2/119 (1.7%)	2 discordant: *3/*17 Spartan but *1/*3 SNP *1/*17 Spartan but *1/*1 SNP	None	[33]
NCT01718535	Spartan FRX	0/325 (0%)	None	None	[34]
Petrek et al. 2016	Spartan RX	0/53 (0%)	None	None	[35,43]
Roberts et al. (2012)	Spartan RX	1/187 (0.5%)	One incorrectly classified as *2 carrier on Spartan	1 FP	[36]
So et al. (2016)	Spartan RX	2/102 (2%)	No details	2 FP	[41]
Wirth et al. (2016)	Spartan RX	1/35 (2.9%)	One incorrect classification as *2/*2 Spartan vs one 2* on Taqman and on GenID	None	[37,44]
Voicu et al. (2024)	Genomadix Cube	0/167 (0%)	None	None	[40]
Meng et al. (2021)	GMEX System	0/408 (0%)	None	None	[39]
Genedrive (2023)	Genedrive *CYP2C19* ID Kit	4/250 (0.8%)	4 discordant: 2 had one LOF on MassARRAY and 2 LOF on Genedrive 2 had two LOF on MassARRAY and 1 LOF on index test	None	[38]

FP: False positive; FN: False negative.

### 3.7. Technical characteristics (e.g. ease of use, test failure rate) of *CYP2C19* POCTs

Twenty-two studies, in 67 reports, provided data on the technical characteristics of the included POCTs [32,33,35–52]. This includes two studies that reported data from a pre-trial and main trial, which were eligible for inclusion and therefore treated as separate studies [32,50]. Ten of the 11 studies that provided data on test accuracy also provided data on the technical characteristics of the test. Twenty studies were published as journal papers, one study was reported only as a conference abstract [48] and one study was reported in an “ID Kit and Performance Data brochure” provided by Genedrive [38]. All studies were reported in English. Table 3 summarizes the characteristics of studies that evaluated technical characteristics of the tests. Full study details are reported in Supplementary Material Section 4.

**Table 3.** Characteristics of studies reporting on the technical characteristics of *CYP2C19* POCT.

	Genomadix (Spartan) *CYP2C19* tests	Genedrive *CYP2C19* ID kit	GMEX system
# studies	19	2	1
Reference	[32,33,35–37,40,41,45–50,52,53,54,55]	[38,51]	[39]
Version of test	**16** Spartan RX **2** Genomadix test (version unclear) **1** Genomadix Cube	**1** Current Genedrive test **1** Earlier version of Genedrive test	**1** GMEX system
Population	**9** PCI **1** Healthy people **1** Not reported **1** Volunteers/control samples **1** Ischemic stroke **1** STEMI **1** Diagnostic coronary angiography **1** Catheterisation **1** Cardiology/angiology/neurology patients **1** Acute coronary syndrome **1** Patients of participating community pharmacies, on P2Y12 inhibitor-based antiplatelet therapy (not previously *CYP2C19* genotyped)	**1** Donor specimens **1** Not reported	**1** Healthy people/people with cardio- and cerebro-vascular diseases
Country	**6** USA **2** Canada **1** Saudi Arabia **1** South Korea **1** Poland **2** International (Canada/US/Mexico/South Korea) **2** the Netherlands **1** Europe (Italy/Netherlands/Belgium) **1** Malta **1** Switzerland **1** Czech Republic	**1** Not reported **1** UK	**1** China
Funding	**1** Industry – test manufacturer **11** Non-industry **1** Industry – other **2** Mixed (industry and non-industry) **1** Not funded **1** Not reported (but Spartan provided genotyping tests) **2** Not reported	**1** Industry – test manufacturer **1** Not reported	**1** Non-industry
Alleles targeted	**13** 2*, 3* and 17* 4 2* 1 2*, 3* 1 2*, 17*	**1** *2, *3, *4, *8, *35, *17 **1** *2,*3,*4,*4b,*10, *17	**1** 2*, 3* and 17*
Who administered POCT	**10** Not reported **1** Onsite testing staff **2** Trial nurses **1** Clinical pharmacist researcher **1** Lab staff/local investigator or nurse **1** Lab staff only **1** Staff physicians **1** Patients (due to COVID-19 pandemic) or pharmacy staff **1** Nurses or physicians	**2** Not reported	**1** Doctors, nurses, clinical researchers

Note: Two reports for the Genomadix (Spartan) *CYP2C19* tests providing technical characteristics data contain details pertaining to an eligible pre-trial and main-trial, which have been counted as separate studies in this review.

PCI: Percutaneous coronary intervention; STEMI: ST-segment elevation myocardial infarction.

One study provided information about the technical characteristics of the GMEX system (the same study that provided accuracy data for this test) [39]. Two studies reported on technical characteristics of the Genedrive *CYP2C19* ID Kit: one evaluated the current version of the test and also provided accuracy data [38], and one evaluated a previous version of the test and only provided data on technical characteristics [48]. All other studies evaluated Genomadix (Spartan) *CYP2C19* tests: one the Genomadix Cube [40], two a Genomadix *CYP2C19* test (version unclear) [51,52] and 16 the Spartan RX test.

As previously noted, the GMEX system study was funded by non-industry organizations. One study of the Genedrive *CYP2C19* ID Kit was funded by the test manufacturer [38], and the other did not report funder details [48]. The Genomadix Cube study was not funded. Of the two studies that evaluated Genomadix *CYP2C19* test (version unclear), one was funded by a mix of industry and non-industry sources [52], and one did not report funder details but noted that Spartan provided the genotyping system and kits). Of the 16 studies that evaluated the Spartan RX test, 11 were funded by non-industry sources, two by industry (one test manufacturer [36]; one by other industry organizations [37]), one was funded by a mix of industry and non-industry sources and two did not report funder details.

Study populations and locations varied between studies. One study of the Genedrive *CYP2C19* ID Kit was undertaken in the UK (no population details reported) [48], and the other Genedrive study tested adult donor specimens (no location reported) [38]. The study of the GMEX system enrolled healthy people and people with cerebrovascular and cardiovascular diseases in China. The study of Genomadix Cube recruited cardiology, angiology and neurology patients in Switzerland [40]. Of the two studies that evaluated Genomadix *CYP2C19* test (version unclear), both were based in the Netherlands; one recruited acute coronary syndrome patients [52] and one recruited adult patients of participating community pharmacies who were taking P2Y12 inhibitor-based antiplatelet treatment with either ticagrelor/prasugrel, and who had not been *CYP2C19* genotyped before [51]. The 16 studies that evaluated Spartan RX included patients undergoing PCI/diagnostic coronary angiography/catheterisation, healthy volunteers, stroke patients, STEMI patients and control samples. One study did not report population [45]. Four of the Spartan RX studies were located in Europe, eight in North America, one in South Korea, one in Saudi Arabia and two studies (reported in the same paper) were multi-national and undertaken in USA/Canada/South Korea/Mexico. Most studies were hospital-based but one study was set within community pharmacies [40].

### 3.8. Test failure rate

Ten studies reported data regarding test failure rate (Table 4). One of these evaluated the GMEX System [39], one evaluated the Genedrive *CYP2C19* ID Kit [38] and eight evaluated Genomadix (Spartan) *CYP2C19* tests. Of these, one evaluated a Genomadix *CYP2C19* test (version unclear) [51] and seven evaluated the Spartan RX test [32,35,37,43,44,49,50].

**Table 4.** Test failure rate results.

Study details	Test	Patients with unavailable result	Missing result details	Action post-test failure	Ref.
Baudhuin et al. (2022)	Spartan RX test	172/2642 (7%)	Main trial: 54/2642 (2%) no result available (no definition); 118 (4%) inconclusive results	Not reported	[32,42,56]
Bergmeijer et al. (2014)	Spartan RX test	39 (8%)	Results inconclusive	Sample was sent to central lab for Taqman testing (30 patients); repeated Spartan test (2 patients); no further genotyping (7 patients).	[46]
Cavallari et al. (2018)	Spartan RX test	129/931 (14%)	56 inconclusive results, 73 device errors	One more sample taken(113 patients), two more samples taken (10 patients), refused recollection of sample (6 patients). 9/123 patients with sample recollection had multiple inconclusive results.	[47]
Petrek et al. 2016	Spartan RX test	7/53 (13.2%)	Failure during amplification process (n = 4), result inconclusive (n = 3) – results not included where only 2/3 alleles gave result	Not reported	[35,43]
Koltowski et al. (2017)	Spartan RX test	2/16 (12.5%)	Results inconclusive	Genotyping repeated but no further information provided.	[52]
Wirth et al. (2016)	Spartan RX test	5/35 (14.3%)	4 tests resulted in error (11.4% – no further details); 1 test inconclusive	Tests resulting in error repeated with new test in line with manufacturer's instructions. Inconclusive test not repeated (patient discharged – no further information provided).	[37,44]
Zhou et al. (2017)	Spartan RX test	25/342 (7.3%)	Main trial: 14 inconclusive results (4%), 10 failed controls (3%), 1 instrument failure (0.3%) (no further information given).	12 patients resulted after re-testing; one refused sample recollection and one had 2 consecutive inconclusive results. No further information provided.	[53,57]
Levens et al. (2023)	Genomadix test (version unclear)	5/144 (3.5%)	Five patients had “erroneous test results” at first attempt.	Two excluded from the study (one withdrew, the other had two consecutive inconclusive results), two had successful result on second run and one had a successful result on third run.	[58]
Meng et al. (2021)	GMEX System	0/480 (0%)	No patients retested or excluded due to incorrect operation or first-run test failure in GMEX group.	Not applicable	[39]
Genedrive (2023)	Genedrive *CYP2C19* ID Kit	0.6% (2/360)	Test failure rate 0.6% (2/360), but it unclear whether test failures were based on donor or contrived samples and whether for initial test run only.	Not reported	[38]

The GMEX System study reported no test failures (0/480 patients). The Genedrive *CYP2C19* ID Kit study reported the test failure rate as 0.6% (2/360), however limited details were provided and it was unclear whether the result was solely for the initial run or after re-testing failed samples. Test failure rate varied between studies that evaluated the Spartan RX test, from a minimum of 7% (172/2642 tests) [32] to a maximum of 14.3% (5/35 patients) for the first test run [37]. Some studies retested samples that had failed, and some then gave results. Terminology used to define what we considered to be test failure rate varied; some studies reported “inconclusive results”, while others detailed “device errors”, “failure during the amplification process” and “not identifying a genotype”. Most of the studies that reported action taken after test failure said that they repeated the test and emphasized the need to take this into consideration when appraising the cost of genotyping.

The study of the Genomadix *CYP2C19* test (version unclear) [51] reported that five out of 144 patients (3.5%) had “erroneous test results” at first attempt. Of these five patients, two were excluded (one withdrew, the other had two consecutive inconclusive results), two had a successful result on the second run and one had a successful result on the third run.

### 3.9. Time to results

Information concerning time to results was reported in 19 studies (Table 5). Of these studies, one reported results for the GMEX System [39], two for Genedrive [38,48] and the other 16 for Genomadix (Spartan) *CYP2C19* tests (of which, one evaluated Genomadix Cube [40]) [33,35–37,40–44,46,47,49–52].

**Table 5.** Time to results data.

Study details	Time to results	Ref.
Al-Rubaish et al. (2021)	First 50 patients: 90–120 min to complete the results	[45]
Azzahhafi et al. (2023)	Median turnover time 5.7 h (IQR: 2.0–12.6), with 90.5% of test results known within 24 h and 96.9% within 48 hr.	[54]
Bergmeijer et al. (2014)	Result within 1 h after collection of buccal swab	[46]
Cavallari et al. (2018)	For all patients genotyped: Median genotype test turnaround time 96 min (interquartile range of 78–144)	[47]
Choi et al. (2016)	**Description of feature of the test**: sample to result took ∼60 min	[33]
Franchi et al. (2020)	Allele status provided within 1 hr. Available when the decision on oral P2Y12-inhibiting therapy most commonly occurs	[49]
Gurbel et al. (2024)	Results available within 1 hour and the results were available immediately for the decision making for the cardiologist.	[50]
Koltowski et al. (2017)	Genotyping took 60 min	[52,59]
Levens et al. (2023)	Average turnaround time ∼75 min. This included pre-test counselling, buccal swap sample collection, hands-on genotyping and 1 h runtime of the device.	[58]
Petrek et al. 2016	Turnaround time (from buccal swab sampling to result print-out): 60 min	[35,43]
Roberts et al. (2012)	Within 60 min from test activation	[36]
So et al. (2016)	Within 55 min of test carrier status for all alleles was available	[41]
Voicu et al. (2024)	117 (70.1%) of Genomadix Cube results were available for treatment decisions within 24 h, and the remaining 50 (29.9%) were available within one week.	[40,60]
Wirth et al. (2016)	Collection of sample to genotyping result within 1 h	[3]
Zhou et al. (2017)	**Description of feature of the test** (pre trial and main trial): results are returned in one hour turnaround time	[53,57]
McDermott et al. (2020) – Genedrive *CYP2C19* ID Kit	**Description of feature of the test**: ∼40 min	[51,61]
Genedrive (2023) – Genedrive *CYP2C19* ID Kit	The time from specimen collection to result was reported as 69 min plus an additional 3 min assay set up time.	[38,62]
Meng et al. (2021) – GMEX System	Average workflow length was 85.0 min (IQR: 85.0–86.0). Results available ∼1.5 h after sample collection (faster than laboratory-based genotyping (2–3) days). Time of sample-to-start, start-to-end and end-to-reports: 6.0 (IQR: 5.0–6.0), 62.0 (IQR: 61.5–62.0) and 18.0 (IQR: 18.0–18.0) min, respectively.	[39]

Fifteen of the 19 studies reported time to result data based on experience from their own study. The one study of the GMEX System reported that POCT results were available approximately 1.5 h after sample collection [39]. One study of Genedrive reported that the time from specimen collection to result was 69 min plus 3 min set up time [38]. Studies that evaluated Genomadix (Spartan) *CYP2C19* tests mostly reported that the time to result was 1 hour [33,35–37,41,43,46,49]. However, one reported it took ∼75 min [51], one reported approximately 90 min [44] and one reported between 90 and 120 min [42]. One study reported that most results were available within 24 h (Genomadix Cube) [40], and one reported that the median turnover for results was 5.7 h (Genomadix test (version unclear)) [52].

Four studies reported time to result data as a description of a feature of the test, rather than as a finding of the study itself [33,48,50]. Three (two of which are a pre-trial and main-trial by the same author [50]) reported that the time from sample to result for the Spartan RX system is 60 min, and one study reported that Genedrive (although we note it is an earlier version of the Genedrive *CYP2C19* ID Kit) is ‘rapid’, taking around 40 mins (no further information reported) [48].

### 3.10. Ease of use of test

Information about the ease of use of the test was reported by 11 studies (Table 6). One study evaluated the GMEX System [39], one evaluated Genedrive [48] and nine evaluated Genomadix (Spartan) *CYP2C19* tests. Of these, eight evaluated the Spartan RX system [32,35–37,43–45,49], and one evaluated a Genomadix test (version unclear) [51].

**Table 6.** Overview of results of ease of use of test.

Study details	Ease of use of test	Ref.
Baudhuin et al. (2022) Test: Spartan RX	Pre-trial: Non laboratory trained personnel successfully conducted rapid genotyping in a POC setting	[32,42]
Bergmeijer et al. (2014) Test: Spartan RX	**Description of feature of the test:** Buccal swab was deemed to be more patient friendly than venepuncture for blood sample, but test only identifies *2, *3, *17, for one patient at a time per genotyping device.	[46]
Cavallari et al. (2018) Test: Spartan RX	The test could not be conducted as a POCT because of the absence of a licensed molecular medical technologist so it had to be sent to central laboratory (the case for all of USA). Also, only a single sample could be genotyped at a time which limited the amount of patients that could be genotyped.	[47]
Davis et al. (2020) Test: Spartan RX	**Description of features of the test:** Barriers to implementation included time constraints, storage and sample stability, personnel requirements/coordination, samples unable to be collected by bedside nurses, patients unable to provide samples, and sample recollection due to interference or improper techniques	[48]
Levens et al. (2023) Test: Genomadix test (version unclear)	Survey to patients in the study who were taking P2Y12 inhibitor-based antiplatelet therapy and who had been genotyped (n = 119): Mainly positive about the use of POCT in community pharmacy and reported that it is convenient, easy, and had increased their understanding of the testing. They recognized the benefits of testing when on medication, thought it added value, they had confidence in the pharmacist in conducting and interpreting the test and felt communication was clear. Survey to pharmacists from the study (n = 14): Facilitators to POCT use in community pharmacy included no resistance from patients, the added value of testing, and that it is the future. Barriers included: poor accessibility and lack of cardiologist cooperation, lack of knowledge and education of pharmacists, testing being a difficult subject for patients, lack of reimbursement, costs, limited time and staff. Survey to cardiologists from the study (n = 8): Mainly felt the testing added value but three indicated they need assistance to implement it. Facilitators included personalized medicine being beneficial and the willingness to reduce costs due to use of ticagrelor and prasugrel. Barriers to implementation included: insufficient knowledge, being unsure if results will affect prescribing policy, increased administrative burden if results not immediately in patient record, and the time burden.	[58]
Petrek et al. 2016 Test: Spartan RX	Simple and non-invasive	[35,43]
Roberts et al. (2012) Test: Spartan RX	After a 30 minute training session, nurses with no previous laboratory training implemented test	[36]
Koltowski et al. (2017) Test: Spartan RX	POCT feasible and convenient. Can be performed by physician or nurse after short training and results achieved quickly.	[52]
Wirth et al. (2016) Test: Spartan RX	Simple procedure, portable, convenient, no laborious preparation, minimal training required to conduct test. User-friendly interpretation with no training required. Storage conditions limit ease of use.	[37,44]
McDermott et al. (2020) Test: Genedrive *CYP2C19* ID Kit	**Description of features of the test:** Portable, no cold chain, simple read out for non-specialist users, rapid (∼40 mins).	[51,61]
Meng et al. (2021) Test: GMEX System	**Description of features of the test:** Easily operated, transmitted and analyzed and presents an effective, user-friendly and simple genotyping approach. It is in line with the affordable, sensitive, specific, user-friendly, rapid and robust, equipment-free and deliverable to end-users WHO-criteria.	[39]

Six studies reported information about the ease of use of the Spartan RX test based on experience from their own study [32,35–37,44,49]. These studies suggested that the test was easy to use, and that it can be undertaken by staff who have had minimal training. One study commented on the tests' ease of interpretation [37]. Reported limitations included storage conditions of the POCT [37], and that only one sample can be genotyped at a time [44].

One study provided survey data from patients, pharmacists and cardiologists about the use of a Genomadix test (version unclear) within community pharmacies in the Netherlands [51]. Patients were mostly positive about the use of POCT in community pharmacy and pharmacists and cardiologists reported barriers and facilitators (Table 6).

Four studies reported data for ease of use as a description of a feature of the test instead of as a study finding [39,43,45,48]. Two of these studies reported on Spartan RX and concur with the aforementioned findings for this test, but added further limitations that the test only detects *2/*3/*17 alleles [43] and that issues with sample collection can take place (e.g. interference leading to sample recollection) [45]. The study that evaluated the GMEX System described the test as user-friendly, effective, rapid and easy to operate, transmit and analyze [39]. Last, the study that evaluated Genedrive reported that the test is simple (with a “simple read out for non-specialist users”), portable, rapid, and does not need analytes to be frozen [48].

### 3.11. Cost of testing

Only three studies reported on the cost of testing. One study evaluated Spartan RX [37] and estimated the cost per patient of the test at 225 euros. The Taqman laboratory assay was estimated to cost 13 euros and the GenID laboratory assay was estimated at 23 euros. The authors do not provide information to explain how they calculated this costing. Two studies evaluated a Genomadix test (version unclear) [51,52]. One of these studies reported that the cost per POCT genotyping analysis (disposable and employee costs) was 150 euros, while the cost per laboratory-based test was 75 euros (machine and employee costs) [42]. The other study, set in community pharmacies, reported that the test materials cost 140 euros, plus 10 euros for the pharmacist time [51].

## 4. Discussion

This review identified 11 studies that provided accuracy data for the *CYP2C19* POCTs in scope: Genomadix (Spartan) *CYP2C19* tests, the Genedrive *CYP2C19* ID Kit and the GMEX system. Overall, the studies estimated that the POCTs are highly accurate for the identification of the specific *CYP2C19* LOF alleles that they are programmed to detect. Nine studies evaluated the accuracy of Genomadix (Spartan) *CYP2C19* tests (seven Spartan RX; one Spartan FRX; one the “Genomadix Cube”). The studies reported very high accuracy (100% summary sensitivity and specificity) for the identification of *2 and/or *3 LOF alleles. There was no sign of a difference across the versions of the test evaluated and the studies were judged to be at low risk of bias. Only one study provided accuracy data for the GMEX System; it was also judged at low risk of bias and it reported that the test had very high accuracy (100% sensitivity and specificity) for the detection of *2 and *3 LOF alleles. Likewise, only one study reported accuracy data for the Genedrive *CYP2C19* ID Kit and it reported that it had very high accuracy (100% sensitivity and specificity) for the detection of *2, *3, *4, *8, *35 alleles. This study was judged at an unclear risk of bias and it was funded by the test manufacturer. Populations varied: the studies of Genomadix (Spartan) *CYP2C19* tests mainly recruited patients undergoing PCI, the GMEX System study recruited healthy people and people with cardiovascular and cerebrovascular diseases and the Genedrive study tested donor specimens. As the GMEX System and the Genedrive *CYP2C19* ID Kit have only been evaluated by one study each, these results should be interpreted with caution, and further research is required to confirm test accuracy of these tests and of the Genomadix Cube (the most current version of the test, also evaluated by one study).

Twenty-two studies reported on the technical characteristics of the included POCTs: one evaluated the GMEX system, two evaluated the Genedrive *CYP2C19* ID Kit (one evaluated a previous version of the test), and all other studies evaluated Genomadix (Spartan) *CYP2C19* tests (one the Genomadix Cube, two a Genomadix *CYP2C19* test (version unclear) and 16 the Spartan RX test). Most studies were hospital-based, but one study was set within community pharmacies [40]. Overall, the tests were reported to be easy to operate and the pharmacy-based study reported that the use of POCT appears to be feasible within community pharmacies. The tests took a similar amount of time to produce results: approximately 1 h for Genomadix (Spartan) *CYP2C19* tests, 90 min for the GMEX system and 69 min for the Genedrive *CYP2C19* ID Kit. The Genedrive *CYP2C19* ID Kit has a potential advantage over the Genomadix (Spartan) *CYP2C19* tests because it does not need to be stored between -15°C and -80°C and used within 15 min of removal from the freezer. The Genedrive test also supports data being uploaded directly into patient records, which is helpful to maximise continuity of care, whereas data is only stored locally within the Genomadix test. Additionally, the Genedrive test displays diplotype plus metabolizer status, which is likely to make interpretation easier, whereas Genomadix reports diplotype only. How the GMEX System is stored, how it integrates with patient records, and what the test reports, is unclear. Questions also remain about the test failure rate and cost of these POCT, which needs to be explored in future studies. There was unexplained variation in test failure rate for Genomadix (Spartan) *CYP2C19* tests (ranging from 3.5% to 14.3%) and only one study each provided test failure data for the other tests. Similarly, only three studies provided cost data and there was no cost information for Genedrive or the GMEX System. However, economic modelling studies have found that POCT testing is likely to be cost-effective for stroke/TIA patients [7,53–56,58].

If further research confirms that the POCTs do have equivalent accuracy to each other, then the decision around which test to consider implementing in practice may be guided by the technical characteristics of the tests outlined above, in addition to their availability for use and the specific LOF alleles that each test can detect. The tests have differing marketing authorization: Genomadix (Spartan) *CYP2C19* tests are CE-IVD and FDA certified, Genedrive is UKCA certified and the company expects to receive CE IVD certification within 12 months, and the GMEX System is reported to be “the only *CYP2C19* POCT marketable in China” [39], but it is unclear whether it has marketing authorization elsewhere. With regards to allele detection, the Genedrive *CYP2C19* ID Kit can detect more *CYP2C19* alleles than the other POCTs (*4, *8 and *35, in addition to *2, *3 and *17). This may provide an advantage, particularly in populations where these alleles are found at higher frequencies. However, *2 and *3 are the most common LOF alleles and the presence of *4 and *8 is low [2]. It is not clear which alleles should be targeted and evidence is still evolving in this area, with alleles such as *9 and *10 currently categorized as “indeterminate” or “likely LOF” [2]. It is therefore possible that further research will identify other important LOF alleles to test for.

In addition to uncertainties around which alleles to target, it is also unclear how treatment should be tailored based on *CYP2C19* testing, whether clinicians are ready and willing to use the results of *CYP2C19* POCTs in practice, and whether the electronic health record is robust enough to allow for the documentation and interpretation of *CYP2C19* status throughout the healthcare system. Before *CYP2C19* POCT could be implemented in the care pathway, there are also a number of governance issues that would need to be considered, such as developing operating procedures (e.g. for recording test results) and information governance strategy, and ensuring sufficient staff knowledge and skills for POCT use [57]. Further barriers highlighted by pharmacists and cardiologists within the study that implemented a Genomadix test (version unclear) within community pharmacies included lack of reimbursement and costs associated with testing, limited time and insufficient staff. Additionally, some countries have regulatory limitations which prohibit the use of POCT near-patient, instead meaning the POCT would have to be conducted at a specially certified genetic laboratory, which may reduce some of the potential benefits of this technology [59].

Despite these uncertainties, it seems likely that the use of *CYP2C19* POCTs would have implications for clinical practice, allowing for faster retrieval of *CYP2C19* genetic results, to be used to guide treatment decisions. As results are provided more quickly by POCT, it may reduce the likelihood that patients are discharged on clopidogrel while awaiting the results of *CYP2C19* laboratory-based tests. Patients with *CYP2C19* LOF may be more likely to be treated with alternative antiplatelet therapies (e.g. ticagrelor/dipyridamole), which could provide more benefit than treatment with clopidogrel [8–11].

Although the use of *CYP2C19* POCTs has the potential to be an improvement on laboratory-based testing, there is intensifying debate around whether reactive testing for a single gene is appropriate, or whether a pre-emptive panel-based approach should be adopted instead [60]. Genetic panel testing would involve individuals being tested for a range of variants across several genes at once, with results readily available in the patient electronic medical record to inform future treatment decisions. This approach is likely to be more cost-effective than testing for a single gene and it would mean that the clinician at the point of care does not need to wait to receive test results. A recent study found that it was feasible to implement genetic panel testing across different European healthcare systems and that doing so reduced the incidence of clinically relevant adverse drug reactions [61]. Clinical experts suggest that genetic panel testing is likely to become more widely used in the next 5–10 years [62], therefore potentially reducing the long-term appropriateness of the implementation of *CYP2C19* POCTs.

Strengths of this review include that it was undertaken in line with guidance on systematic review conduct and it is reported according to PRISMA-2020 and PRISMA-DTA. We also followed clear inclusion criteria as specified in our pre-registered protocol [27]. Our review methodology was thorough and transparent, and two reviewers were involved in each step of the review, including data extraction and risk of bias assessment, using the validated QUADAS-2 tool. We undertook extensive searches, contacted test developers, and received guidance from laboratory colleagues to ensure that all relevant *CYP2C19* POCTs, and available literature evaluating the POCTs, were included in the review. Overall, we found that the terminology used to refer to POCTs is inconsistent within the literature and tests are sometimes referred to as a POCT incorrectly. This made it challenging to decipher if a test being evaluated in a primary study was truly a POCT that is intended to be used outside of a conventional laboratory setting. Additionally, the amount of accessible information about the POCTs online from test developers, including test properties, marketing authorization and indication for use, varies. For example, Genedrive and Genomadix provide details of test properties and related research on their websites, whereas we identified limited information about the GMEX System, beyond what is reported in the included journal articles. Discussions with our clinician co-author and laboratory colleagues were therefore important to inform inclusion decisions.

A further strength of this review is that although we dichotomized results in this review into the presence and absence of LOF alleles, we report all discordant test results, allowing the reader to easily interpret the data in other ways. A potential limitation is that, in all studies, the laboratory-based reference standard only tested for the same alleles as the POCT and it is therefore unclear whether accuracy would have differed if the reference standard detected any LOF allele. However, this may have little impact on findings because the included POCTs do test for the most common LOF alleles.

There are a number of areas in which further research is required. As noted, there is a lack of data on the GMEX System and the Genedrive *CYP2C19* ID Kit, and only one study provided data on the Genomadix Cube *CYP2C19* system (the most current version of this POCT). Therefore, further studies that provide data on the accuracy and technical characteristics of these tests, outside of the laboratory, are required. Additionally, studies in this review employed various laboratory-based tests as the reference standard (assumed to have 100% accuracy), but there is no clear single reference standard, so it is not possible to stipulate a reference standard for evaluation of laboratory based tests. In future it would be interesting to compare the accuracy of both *CYP2C19* POCTs and laboratory tests to P2Y12-pathway specific platelet function tests (phenotype test) as a reference standard, or to explore the potential to use these tests as an alternative to genetic testing. Additionally, tests exist (for example the LaCAR *CYP2C19* test [63]) that are said to be somewhere between a POCT with fast results and high cost per test, and a laboratory-based test with slower results and lower cost. Future research could explore the accuracy and technical characteristics of such tests, which are designed for use in the laboratory, and investigate their properties relative to *CYP2C19* POCT.

## 5. Conclusion

In conclusion, the existing evidence suggests that *CYP2C19* POCTs have very good accuracy and ease of use, and may have the potential to provide results faster than laboratory-based testing. However, there is limited evidence available for the GMEX System and the Genedrive *CYP2C19* ID Kit, and there are uncertainties related to whether the implementation of *CYP2C19* POCTs in clinical practice is appropriate in the changing testing landscape. Further accuracy data are required on the GMEX System and the Genedrive *CYP2C19* ID Kit, and more data on test failure rate and cost are needed for all tests.

## Supplementary Material

Supplementary Materials
