# Supplementary material for: Accuracy and technical characteristics of CYP2C19 point of care tests: a systematic review
Source: Pharmacogenomics. 2024 Sep 4;25(8-9):407–23. doi: 10.1080/14622416.2024.2392479 (PMC11418221; doi:10.1080/14622416.2024.2392479)
Supplement: Supplementary Materials [file IPGS_A_2392479_SM0001.docx]

Supplementary material

# Section 1: Characteristics of CYP2C19 point of care and laboratory-based tests

Note: all of the included POCT use a buccal sample

| Name of test | General information | *CYP2C19* alleles targeted |
| --- | --- | --- |
| Genomadix Cube *CYP2C19* system | - *In vitro* diagnostic test produced by Genomadix Incorporated, used to detect alleles of the *CYP2C19* gene from genomic DNA obtained from a buccal sample. - The test kit cartridges must be stored between −15°C and −80°C and used within 15 minutes of removal from the freezer. - It is intended to be used in conjunction with clinical judgement and routine monitoring to determine therapeutic strategy for drugs metabolized by the *CYP2C19* enzyme. - Results are stored locally on a laptop connected to the device and can be exported as a PDF. - CE IVD and FDA certification. | *2, *3, *17 |
| Genedrive *CYP2C19* ID Kit | - *In vitro* diagnostic molecular assay produced by Genedrive Diagnostics, used to detect alleles of the *CYP2C19* gene from genomic DNA obtained from a buccal sample. - The ID Kit is used in conjunction with the Genedrive System to provide a result of *CYP2C19* genotypes. It should be stored between 2°C and 30°C. - It is intended to be used by healthcare professionals, located in a near-patient environment, and it is indicated for use as an aid to clinicians in determining therapeutic strategies. - Automated data analysis is performed by the Genedrive system and metaboliser status result is displayed on the screen to the user. Results will be able to be transferred electronically to patient records by internet or through third-party middleware, or printed with an optional label printer. - UKCA marking certification achieved, and CE IVD expected within <12 months. | *2, *3, *4, *8, *17, *35 |
| GMEX system | - Portable DNA analyser, genotyping reagents and a buccal sample collection kit, produced by Chongqing Jingyin Bioscience. - The GMEX system integrates automated PCR-based amplification, fluorescent signal detection and genotype determination. It is conducted near-patient by trained doctors, nurses or clinical researchers. - “Marketed by Chongqing Jingyin Bioscience” “widely used in emergency, outpatient and rapid clinical diagnosis”(1) | *2, *3, *17 |
| Sanger *CYP2C19* sequencing | Sequences a single DNA fragment at a time. The time to run the test depends on sample numbers and number of alleles tested (more = longer time). | All alleles |
| Next-generation *CYP2C19* gene sequencing | Sequences millions of short DNA sequences in parallel. Quicker turnaround for large sample numbers compared to Sanger sequencing. | All alleles |
| Targeted *CYP2C19* gene variant detection | Targeted genotyping assay amplifies and detects specific variants in target genomic DNA. Examples include:   - Polymerase chain reaction (PCR)-based SNP genotyping assays using fluorescent reporter systems, such as TaqMan (ThermoFisher) - Other PCR-based genotyping panels that use proprietary detection methods, such as the xTAG *CYP2C19* Kit v3 (Luminex) - Variant detection using mass spectrometry, such as MassARRAY (Agena Bioscience) - Loop-mediated isothermal amplification (LAMP), such as the LAMP human *CYP2C19* mutation KIT (LaCAR MDx Technologies)   The methods of detection, equipment requirements and throughput capability vary between systems. | Potential to target all alleles but usually target specific alleles. |

# Section 2: Search strategy

## Main search

**Search Purpose:** to identify studies reporting test accuracy or technical performance data (including data on costs) for point of care tests which detect the presence of *CYP2C19* loss of function alleles.

**Database:** MEDLINE (MEDALL)

**Host:** OVID

**Data Parameters:** 1946 to present

**Date of search**: 4 May 2023

| **Search Strategy** | **Search Narrative** |
| --- | --- |
| 1     Point-of-Care Testing/ (3932)  2     ((Point of Care adj2 (test* or genotyp* or assay)) or POCT or POC).ti,ab,kf,kw. (16638)  3     (Genomadix* or Spartan* or Genedrive* or Verigene* or NanosphereVerigene* or GMEX* or Q3).af. (7536)  4     1 or 2 or 3 (25866) | **Search for Point of Care Tests**  In lines 1-3 we search for Point of Care Tests. We search for the tests we know specifically to be in scope, following clinical and technical input to the search approach and protocol (namely those listed at Line 3). We search, also, generally on terms for point of care tests at Lines 1 and 2. The purpose of Lines 1 and 2 are to identify studies evaluating tests that we are unaware of, as well to identify tests we are aware of but which do not report the test name in the title or abstract of the study report.  Line 1 is the indexing (MeSH) term for Point of Care Testing. That this is an indexing term is represented by /.  Line 2 takes general descriptive terms for point of care tests and acronyms. We search in the following fields:  ti = title;  ab = abstract;  kf = key field (free-text terms chosen by authors to describe their study); and  kw = key word.  Line 3 searches for the tests we believe to be in scope. We anticipate that this line duplicates Line 2, so it is included for sensitivity, as explained above. This search is in ‘all fields’ (.af.). The most sensitive search available in the Ovid interface.  We use truncation in this search, indicated by *. This captures alternate word endings as well as where a test name is immediately followed by the trade mark (e.g., SpartanTM or Spartan^tm^) |
| 5     Cytochrome P-450 CYP2C19/ (3433)  6     (CYP2C19* or cypiic19* or "Cytochrome P-450").ti,ab,kw,kf. (20629)  7     *"Loss of Function Mutation"/ (641)  8     5 or 6 or 7 (21816) | **Terms for CYP2C19**    The free-text terms are truncated using the * marker. Truncation ensures that the root word and other possible variations are identified and returned by the search. We have truncated CYP2C19 (using the * marker again) to identify CYP2C19*2, CYP2C19*3, and CYP2C9*17, and other alleles. |
| 9     Clopidogrel/ (10128)  10     (clopidogrel* or clopilet* or duoplavin* or grepid* or inhiplat* or Iscover* or "klogel-A*" or mdco* or myogrel* or osvix* or plavi* or plavitor* or plavix* or pregrel* or zopya* or zylagren* or zyllt* or "R 130964" or "R-130964" or  R130964 or "SR 25990" or "SR-25990" or SR25990 or A74586SNO7 or "113665-84-2" or "120202−65−5" or "120202−66−6"  or "120202−67−7" or "744256−69−7" or "894353−16−3" or "90055−48−4" or "94188−84−8").ti,ab,kw,kf.  (14554)  11     9 or 10 (16475) | **Terms for Clopidogrel**  We search for Clopidogrel using the primary name, brand names, external IDs (e.g., R 130964) and UNII and CAS registry numbers. |
| 12     4 and (8 or 11) (193) | Line 12 combines the search, taking   - POCT terms; AND - terms for CYP2C19 OR - terms Clopidogrel |
| 13     (NCT01761786 or NCT02724319 or NCT02065479 or NCT01930773 or NCT01718535 or NCT01742117 or NCT01742117 or  NCT01184300 or NCT01184300 or NCT01452139 or NCT01107925 or NCT01107912 or NCT01390974 or NCT01994941 or NCT03347435 or  NCT04078737).af. (29) | At Line 13 we search for studies using the Clinical Trials.gov registry number. The purpose of this search is to identify reports of studies we know already to be in scope based on scoping and a recent systemic review for NICE. |
| 14     12 or 13 (214) | Line 14 combines the main search and Line 13 (the search for known study IDs). These are combined using the Boolean connector OR, meaning that we will identify results from the main search (Line 12) OR from the searches for known study reports (Line 13).  The search is not limited by publication date, publication type or study design, or by language of publication. |

## 2024 update search

**Search Purpose:** to identify studies reporting test accuracy or technical performance data (including data on costs) for point of care tests which detect the presence of *CYP2C19* loss of function alleles.

**Database:** MEDLINE (MEDALL)

**Host:** OVID

**Data Parameters:** 1946 to June 18, 2024

**Date of search**: 19 June 2024

| **Search Strategy** | **Search Narrative** |
| --- | --- |
| 1     Point-of-Care Testing/ (4403)  2     ((Point of Care adj2 (test* or genotyp* or assay)) or POCT or POC).ti,ab,kf,kw. (19387)  3     (Genomadix* or Spartan* or Genedrive* or Verigene* or NanosphereVerigene* or GMEX* or Q3).af. (9339)  4     1 or 2 or 3 (30545) | **Search for Point of Care Tests**  In lines 1-3 we search for Point of Care Tests. We search for the tests we know specifically to be in scope, following clinical and technical input to the search approach and protocol (namely those listed at Line 3). We search, also, generally on terms for point of care tests at Lines 1 and 2. The purpose of Lines 1 and 2 are to identify studies evaluating tests that we are unaware of, as well to identify tests we are aware of but which do not report the test name in the title or abstract of the study report.  Line 1 is the indexing (MeSH) term for Point of Care Testing. That this is an indexing term is represented by /.  Line 2 takes general descriptive terms for point of care tests and acronyms. We search in the following fields:  ti = title;  ab = abstract;  kf = key field (free-text terms chosen by authors to describe their study); and  kw = key word.  Line 3 searches for the tests we believe to be in scope. We anticipate that this line duplicates Line 2, so it is included for sensitivity, as explained above. This search is in ‘all fields’ (.af.). The most sensitive search available in the Ovid interface.  We use truncation in this search, indicated by *. This captures alternate word endings as well as where a test name is immediately followed by the trade mark (e.g., SpartanTM or Spartan^tm^) |
| 5     Cytochrome P-450 CYP2C19/ (3608)  6     (CYP2C19* or cypiic19* or "Cytochrome P-450").ti,ab,kw,kf. (21145)  7     *"Loss of Function Mutation"/ (650)  8     5 or 6 or 7 (22345) | **Terms for CYP2C19**    The free-text terms are truncated using the * marker. Truncation ensures that the root word and other possible variations are identified and returned by the search. We have truncated CYP2C19 (using the * marker again) to identify CYP2C19*2, CYP2C19*3, and CYP2C9*17, and other alleles. |
| 9     Clopidogrel/ (10476)  10     (clopidogrel* or clopilet* or duoplavin* or grepid* or inhiplat* or Iscover* or "klogel-A*" or mdco* or myogrel* or osvix* or plavi* or plavitor* or plavix* or pregrel* or zopya* or zylagren* or zyllt* or "R 130964" or "R-130964" or  R130964 or "SR 25990" or "SR-25990" or SR25990 or A74586SNO7 or "113665-84-2" or "120202−65−5" or "120202−66−6"  or "120202−67−7" or "744256−69−7" or "894353−16−3" or "90055−48−4" or "94188−84−8").ti,ab,kw,kf.  (15413)  11     9 or 10 (17364) | **Terms for Clopidogrel**  We search for Clopidogrel using the primary name, brand names, external IDs (e.g., R 130964) and UNII and CAS registry numbers. |
| 12     4 and (8 or 11) (208) | Line 12 combines the search, taking   - POCT terms; AND - terms for CYP2C19 OR - terms Clopidogrel |
| 13     (NCT01761786 or NCT02724319 or NCT02065479 or NCT01930773 or NCT01718535 or NCT01742117 or NCT01742117 or  NCT01184300 or NCT01184300 or NCT01452139 or NCT01107925 or NCT01107912 or NCT01390974 or NCT01994941 or NCT03347435 or  NCT04078737).af. (39) | At Line 13 we search for studies using the Clinical Trials.gov registry number. The purpose of this search is to identify reports of studies we know already to be in scope based on scoping and a recent systemic review for NICE. |
| 14     12 or 13 (238) | Line 14 combines the main search and Line 13 (the search for known study IDs). These are combined using the Boolean connector OR, meaning that we will identify results from the main search (Line 12) OR from the searches for known study reports (Line 13).  The search is not limited by publication date, publication type or study design, or by language of publication. |
| 15 (2023* or 2024*).dt,dp,ed,ep,yr. (2554575)  16 14 and 15 (33) | Lines 15 and 16 apply a date limit to the search so we update from the original search until June 18 2024. |

# Section 3: Included and excluded studies

## Included studies

| **Study** | **References (main report for this review listed first)** |
| --- | --- |
| **Al Rubaish, 2021** | Al-Rubaish AM, Al-Muhanna FA, Alshehri AM, Alsulaiman AA, Alabdulali MM, Alkhamis F, et al. Prevalence of CYP2C19*2 carriers in Saudi ischemic stroke patients and the suitability of using genotyping to guide antiplatelet therapy in a university hospital setup. Drug metabolism and personalized therapy. 2021;37(1). |
| **Azzahhafi, 2023** | Azzahhafi J, Broek WW, Chan Pin Yin DR, Harmsze AM, van Schaik RH, Ten Berg JM. The Clinical Implementation of CYP2C19 Genotyping in Patients with an Acute Coronary Syndrome: Insights From the FORCE-ACS Registry. Journal of Cardiovascular Pharmacology and Therapeutics. 2023 Oct;28:10742484231210704. |
|  | Azzahhafi J, van den Broek W, Chan Pin Yin D, Van Schaik R, Berg JT. TCT-381 The Clinical implementation of CYP2C19 Genotyping in Patients With an Acute Coronary Syndrome: Insights From the FORCE-ACS Registry. Journal of the American College of Cardiology. 2023 Oct 24;82(17_Supplement):B152-. |
| **Baudhuin, 2022**  ***(TAILOR-PCI; NCT01742117)*** | Baudhuin LM, Train LJ, Goodman SG, Lane GE, Lennon RJ, Mathew V, et al. Point of care CYP2C19 genotyping after percutaneous coronary intervention. Pharmacogenomics journal. 2022;22(5). |
|  | Avram R, So D, Iturriaga E, Byrne J, Lennon R, Murthy V, et al. Patient Onboarding and Engagement to Build a Digital Study After Enrollment in a Clinical Trial (TAILOR-PCI Digital Study): Intervention Study. JMIR formative research. 2022;6(6). |
|  | Avram R, Byrne J, So D, Iturriaga E, Lennon R, Murthy V, Geller N, Goodman S, Rihal C, Rosenberg Y, Bailey K. Digital Tool-Assisted Hospitalization Detection in the Tailored Antiplatelet Initiation to Lessen Outcomes due to Decreased Clopidogrel Response After Percutaneous Coronary Intervention Study Compared to Traditional Site-Coordinator Ascertainment: Intervention Study. Journal of medical Internet research. 2023 Nov 10;25:e47475. |
|  | Capodanno D, Angiolillo DJ, Lennon RJ, Goodman SG, Kim S-W, O'Cochlain F, et al. ABCD-GENE Score and Clinical Outcomes Following Percutaneous Coronary Intervention: Insights from the TAILOR-PCI Trial. Journal of the American Heart Association. 2022;11(4). |
|  | Huxley S, Pereira N, Borah B, Lennon R, Moriarty J. Direct cost analysis of genetic testing for CYP2C19∗2 and ∗3 loss of function variants in percutaneous coronary intervention patients. European respiratory journal. 2022;60. |
|  | Ingraham BS, Farkouh ME, Lennon RJ, So D, Goodman SG, Geller N, et al. Genetic-Guided Oral P2Y12 Inhibitor Selection and Cumulative Ischemic Events After Percutaneous Coronary Intervention. JACC Cardiovascular interventions. 2023;16(7). |
|  | Madan M, Abbott JD, Lennon R, So DYF, MacDougall AM, McLaughlin MA, et al. Sex-Specific Differences in Clinical Outcomes After Percutaneous Coronary Intervention: insights from the TAILOR-PCI Trial. Journal of the American Heart Association. 2022;11(12). |
|  | Mathew RO, Sidhu MS, Rihal CS, Lennon R, El-Hajjar M, Yager N, et al. Safety and Efficacy of CYP2C19 Genotype-Guided Escalation of P2Y12  Inhibitor Therapy After Percutaneous Coronary Intervention in Chronic Kidney Disease: a Post Hoc Analysis of the TAILOR-PCI Study. Cardiovascular drugs and therapy / sponsored by the International Society of Cardiovascular Pharmacotherapy. 2022. |
|  | Pereira NL, Avram R, So DY, Iturriaga E, Byrne J, Lennon RJ, et al. Rationale and design of the TAILOR-PCI digital study: Transitioning a randomized controlled trial to a digital registry. American heart journal. 2021;232. |
|  | Pereira NL, Farkouh ME, So D, Lennon R, Geller N, Mathew V, et al. Effect of Genotype-Guided Oral P2Y12 Inhibitor Selection vs Conventional Clopidogrel Therapy on Ischemic Outcomes After Percutaneous Coronary Intervention: The TAILOR-PCI Randomized Clinical Trial. JAMA. 2020;324(8). |
|  | Trial registration: <https://ClinicalTrials.gov/show/NCT01742117> |
| **Bergmeijer, 2018**  ***(POPular Genetics; NCT01761786)*** | Bergmeijer TO, Vos GJ, Claassens DM, Janssen PW, Harms R, der Heide Rv, et al. Feasibility and implementation of CYP2C19 genotyping in patients using antiplatelet therapy. Pharmacogenomics. 2018;19(7). |
|  | Bergmeijer TO, Janssen PW, Schipper JC, Qaderdan K, Ishak M, Ruitenbeek RS, et al. CYP2C19 genotype-guided antiplatelet therapy in ST-segment elevation myocardial infarction patients-Rationale and design of the Patient Outcome after primary PCI (POPular) Genetics study. American heart journal. 2014;168(1). |
|  | Bergmeijer TO, Janssen PWA, Asselbergs FW, Schipper JC, Van 'T Hof AW, Dewilde WJM, et al. A tailored antiplatelet strategy in STEMI patients based on CYP2C19 genotyping is feasible in daily practice-POPular Genetics study. European heart journal. 2014;35. |
|  | Claassens DMF, Bergmeijer TO, Vos GJA, Hermanides RS, van 't Hof AWJ, van der Harst P, et al. Clopidogrel Versus Ticagrelor or Prasugrel After Primary Percutaneous Coronary Intervention According to CYP2C19 Genotype: A POPular Genetics Subanalysis. Circulation Cardiovascular interventions. 2021;14(4). |
|  | Claassens DMF, Gimbel ME, Bergmeijer TO, Vos GJA, Hermanides RS, van der Harst P, et al. Clopidogrel in noncarriers of CYP2C19 loss-of-function alleles versus ticagrelor in elderly patients with acute coronary syndrome: A pre-specified sub analysis from the POPular Genetics and POPular Age trials CYP2C19 alleles in elderly patients. International Journal of Cardiology. 2021;334. |
|  | Claassens DMF, Tavenier AH, Hermanides RS, Vos GJA, Hinrichs DL, Bergmeijer TO, et al. Reloading When Switching From Ticagrelor or Prasugrel to Clopidogrel Within 7 Days After STEMI. JACC: Cardiovascular Interventions. 2020;13(5). |
|  | Claassens DMF, van Dorst PWM, Vos GJA, Bergmeijer TO, Hermanides RS, van 't Hof AWJ, et al. Cost Effectiveness of a CYP2C19 Genotype-Guided Strategy in Patients with Acute Myocardial Infarction: Results from the POPular Genetics Trial. American journal of cardiovascular drugs : drugs, devices, and other interventions. 2022;22(2). |
|  | Claassens DMF, van Dorst PWM, Vos GJA, Bergmeijer TO, Hermanides RS, van 't Hof AWJ, et al. Cost Effectiveness of a CYP2C19 Genotype-Guided Strategy in Patients with Acute Myocardial Infarction: results from the POPular Genetics Trial. American journal of cardiovascular drugs. 2021. |
|  | Tavenier AH, Claassens DMF, Hermanides RS, Vos GJA, Bergmeijer TO, Kelder JC, et al. Efficacy and safety of glycoprotein IIb/IIIa inhibitors in addition to P2Y12 inhibitors in ST-segment elevation myocardial infarction: A subanalysis of the POPular Genetics trial. Catheterization and Cardiovascular Interventions. 2022;99(3). |
|  | Trial registration: <https://ClinicalTrials.gov/show/NCT01761786> |
|  | van den Broek WW, Mani N, Azzahhafi J, Ten Berg JM. CYP2C9 Polymorphisms and the risk of cardiovascular events in patients treated with clopidogrel: combined data from the POPular genetics and POPular AGE trials. American Journal of Cardiovascular Drugs. 2023 Mar;23(2):165-72. |
| **Cavillari, 2018**  ***(NCT02724319)*** | Cavallari LH, Franchi F, Rollini F, Been L, Rivas A, Agarwal M, et al. Clinical implementation of rapid CYP2C19 genotyping to guide antiplatelet therapy after percutaneous coronary intervention. Journal of translational medicine. 2018;16(1). |
|  | Trial registration: <https://ClinicalTrials.gov/show/NCT02724319> |
| **Choi, 2016** | Choi J-L, Kim B-R, Woo K-S, Kim K-H, Kim J-M, Kim M-H, et al. The Diagnostic Utility of the Point-of-Care CYP2C19 Genotyping Assay in Patients with Acute Coronary Syndrome Dosing Clopidogrel: Comparison with Platelet Function Test and SNP Genotyping. Annals of clinical and laboratory science. 2016;46(5). |
| **Davis, 2020** | Davis BH, DeFrank G, Limdi NA, Harada S. Validation of the Spartan RXCYP2C19 Genotyping Assay Utilizing Blood Samples. Clinical and translational science. 2020;13(2). |
| **Franchi, 2020**  ***(NCT02065479)*** | Franchi F, Rollini F, Rivas J, Rivas A, Agarwal M, Briceno M, et al. Prasugrel Versus Ticagrelor in Patients With CYP2C19 Loss-of-Function Genotypes: results of a Randomized Pharmacodynamic Study in a Feasibility Investigation of Rapid Genetic Testing. JACC: basic to translational science. 2020;5(5). |
|  | Franchi F, Rollini F, Rivas J, Rivas A, Agarwal M, Briecno M, et al. Platelet inhibitory profiles of prasugrel versus ticagrelor in patients with CYP2C19 loss-of-function genotypes undergoing percutaneous coronary intervention: results of a randomized feasibility study. European heart journal. 2019;40. |
|  | Trial registration (main): <https://ClinicalTrials.gov/show/NCT02065479> |
|  | Trial registration (sub-study using sample from main): <https://ClinicalTrials.gov/show/NCT03489863> |
| **Genedrive. 2023.** | Genedrive CYP2C19 ID Kit. |
|  | Genedrive CYP2C19 ID Kit: Performance Data for UKCA Marked Device. |
| **Gurbel, 2024** | Gurbel PA, Bliden K, Sherwood M, Taheri H, Tehrani B, Akbari M, Yazdani S, Asgar JA, Chaudhary R, Tantry US. Development of a routine bedside CYP2C19 genotype assessment program for antiplatelet therapy guidance in a community hospital catheterization laboratory. Journal of Thrombosis and Thrombolysis. 2024 Apr;57(4):566-75. |
|  | Gurbel PA, Bell R, Bliden K, Yazdani S, Taheri H, Akbari M, et al. Bedside Testing of CYP2C19 Genotype to Guide Antiplatelet Therapy: Implementation in the Catheterization Laboratory. Journal of the American College of Cardiology. 2018;71(11S):A1202-A. |
|  | Gurbel PA, Bliden K, Sherwood M, Yazdani S, Taheri H, Truesdell A, et al. POINT-OF-CARE CYP2C19 GENOTYPE GUIDED ANTIPLATELET THERAPY IN CARDIAC CATHETERIZATION LABORATORY AND CLINICAL OUTCOMES AFTER PCI. Journal of the American College of Cardiology (JACC). 2019;73(9). |
| **Levens, 2023** | Levens AD, den Haan MC, Jukema JW, Heringa M, van den Hout WB, Moes DJ, Swen JJ. Feasibility of Community Pharmacist-Initiated and Point-of-Care CYP2C19 Genotype-Guided De-Escalation of Oral P2Y12 Inhibitors. Genes. 2023 Feb 25;14(3):578. |
| **McDermott, 2020** | McDermott JH AS, Wright S, Sen D, Miele G, Smith CJ, Payne k , Newman W, editor Development of a Point-of-Care Pharmacogenetic Test for CYP2C19 Allowing Genotype Guided Antiplatelet Prescribing to Prevent Recurrent Strokes2020. |
|  | McDermott J, Ainsworth S, Wright S, Sen D, Miele G, Smith C, et al., editors. Development of a Point of Care Test for CYP2C19Allowing Genotype Guided Antiplatelet Prescribing to Prevent Recurrent Ischaemic Strokes. EUROPEAN JOURNAL OF HUMAN GENETICS; 2020: SPRINGERNATURE CAMPUS, 4 CRINAN ST, LONDON, N1 9XW, ENGLAND. |
| **Meng, 2021**  ***(CHANCE-2; NCT04078737)*** | Meng X, Wang A, Zhang G, Niu S, Li W, Han S, et al. Analytical validation of GMEX rapid point-of-care CYP2C19 genotyping system for the CHANCE-2 trial. Stroke and vascular neurology. 2021;6(2). |
|  | Meng X, Wang A, Xie X, Pan Y, Johnston SC, Li H, et al. Ticagrelor versus Clopidogrel in CYP2C19 Loss-of-Function Carriers with Stroke or TIA. New England Journal of Medicine. 2021;385(27). |
|  | Meng X, Wang A, Tian X, Johnston C, Li H, Bath PM, Xu Q, Zhang Y, Xie X, Jing J, Lin J. One-Year Outcomes of Early Therapy With Ticagrelor vs Clopidogrel in CYP2C19 Loss-of-Function Carriers With Stroke or TIA Trial. Neurology. 2024 Feb 13;102(3):e207809. |
|  | Jing J, Xie X, Johnston SC, Bath PM, Li Z, Zhao X, et al. Genotype-Guided Dual Antiplatelet Use for Transient Ischemic Attack and Minor Stroke by Imaging Status: Subgroup Analysis of the CHANCE-2 Trial. Annals of neurology. 2023;93(4). |
|  | Johnston C, Bath PM, Meng X, Jing J, Xie X, Wang A, et al. Clopidogrel with aspirin in High-risk patients with Acute Non-disabling Cerebrovascular Events II (CHANCE-2): Rationale and design of a multicentre randomised trial. Stroke and Vascular Neurology. 2021;6(2). |
|  | Liu H, Jing J, Wang A, Xu Q, Meng X, Li H, et al. Stroke Recurrence and Antiplatelets in Posterior Versus Anterior Circulation Minor Stroke or Transient Ischemic Attack. Stroke. 2023;54(4). |
|  | Liu H, Jing J, Wang A, Xu Q, Meng X, Li H, Li Z, Wang Y. Genotype-guided dual antiplatelet therapy in minor Stroke or transient ischemic attack with a single small subcortical infarction. Neurology. 2023 Apr 18;100(16):e1643-54. |
|  | Wang A, Meng X, Tian X, Johnston SC, Li H, Bath PM, et al. Effect of Hypertension on Efficacy and Safety of Ticagrelor-Aspirin Versus Clopidogrel-Aspirin in Minor Stroke or Transient Ischemic Attack. Stroke. 2022;53(9). |
|  | Wang A, Meng X, Tian X, Zuo Y, Bath PM, Li H, et al. Ticagrelor Aspirin vs Clopidogrel Aspirin in CYP2C19 Loss-of-Function Carriers With Minor Stroke or TIA Stratified by Risk Profile. Neurology. 2023;100(5). |
|  | Wang A, Xie X, Tian X, Johnston SC, Li H, Bath PM, et al. Ticagrelor–Aspirin Versus Clopidogrel–Aspirin Among CYP2C19 Loss-of-Function Carriers With Minor Stroke or Transient Ischemic Attack in Relation to Renal Function: a Post Hoc Analysis of the CHANCE-2 Trial. Annals of internal medicine. 2022;175(11). |
|  | Trial registration: <https://ClinicalTrials.gov/show/NCT04078737> |
|  | Wang C, Jia W, Jing J, Meng X, Wang A, Xu Q, Zhang X, Pan Y, Xie X, Johnston SC, Bath PM. Ticagrelor Versus Clopidogrel in Minor Stroke or Transient Ischemic Attack With Intracranial Artery Stenosis: A Post Hoc Analysis of CHANCE‐2. Journal of the American Heart Association. 2023 Nov 7;12(21):e031611. |
|  | Wang A, Tian X, Xie X, Li H, Bath PM, Jing J, Lin J, Wang Y, Zhao X, Li Z, Liu L. Differential effect of ticagrelor versus clopidogrel by homocysteine levels on risk of recurrent stroke: a post hoc analysis of the CHANCE-2 trial. CMAJ. 2024 Feb 12;196(5):E149-56. |
|  | Wang A, Tian X, Xie X, Li H, Jing J, Lin J, Wang Y, Zhao X, Li Z, Liu L, Wang Y. Effects of remnant cholesterol on the efficacy of genotype-guided dual antiplatelet in CYP2C19 loss-of-function carriers with minor stroke or transient ischaemic attack: a post-hoc analysis of the CHANCE-2 trial. EClinicalMedicine. 2024 Jan 1;67. |
|  | Xie X, Jing J, Meng X, Claiborne Johnston S, Bath PM, Li Z, Zhao X, Liu L, Wang Y, Xu Q, Wang A. Dual antiplatelet therapies and causes in minor stroke or transient ischemic attack: a prespecified analysis in the CHANCE-2 Trial. Stroke. 2023 Sep;54(9):2241-50. |
|  | Xie X, Jing J, Meng X, Johnston SC, Bath PM, Li Z, Zhao X, Wang Y, Xu Q, Wang A, Jiang Y. Dual Antiplatelet Therapy After Embolic Stroke of Undetermined Source: A Subgroup Analysis of the CHANCE-2 Trial. Stroke. 2024 Jun 11. |
|  | Xie X, Johnston SC, Wang A, Xu Q, Bath PM, Pan Y, Li H, Lin J, Wang Y, Zhao X, Li Z. Association of CYP2C19 Loss-of-Function Metabolizer Status With Stroke Risk Among Chinese Patients Treated With Ticagrelor-Aspirin vs Clopidogrel-Aspirin: A Prespecified Secondary Analysis of a Randomized Clinical Trial. JAMA Network Open. 2023 Jun 1;6(6):e2317037-. |
|  | Zhang J, Wang A, Tian X, Meng X, Xie X, Jing J, Lin J, Wang Y, Li Z, Liu L, Li H. Impact of body mass index on efficacy and safety of ticagrelor versus clopidogrel in patients with minor stroke or transient ischemic attack. CMAJ. 2023 Jul 10;195(26):E897-904. |
|  | Zhang X, Jing J, Wang A, Xie X, Johnston SC, Li H, Bath PM, Xu Q, Lin J, Wang Y, Zhao X. Efficacy and safety of dual antiplatelet therapy in the elderly for stroke prevention: a subgroup analysis of the CHANCE-2 trial. Stroke and Vascular Neurology. 2024 Jan 29:svn-2023. |
| **NCT01718535** | Trial registration: <https://ClinicalTrials.gov/show/NCT01718535> |
| **Petrek, 2016** | Petrek M, Kocourkova L, Zizkova V, Nosek Z, Taborsky M, Petrkova J. Characterization of Three CYP2C19 Gene Variants by MassARRAY and Point of Care Techniques: Experience from a Czech Centre. Archivum immunologiae et therapiae experimentalis. 2016;64. |
|  | Petrkova J, Paskova L, Zizkova V, Nosek Z, Taborsky M, Petrek M, editors. POCT for determination of basic pharmacogenetic profile for individualization of antiplatelet therapy: pilot study. EUROPEAN HEART JOURNAL; 2014: OXFORD UNIV PRESS GREAT CLARENDON ST, OXFORD OX2 6DP, ENGLAND. |
| **Roberts, 2012**  ***(RAPID-GENE; NCT01184300)*** | Roberts JD, Wells GA, Le May MR, Labinaz M, Glover C, Froeschl M, et al. Point-of-care genetic testing for personalisation of antiplatelet treatment (RAPID GENE): a prospective, randomised, proof-of-concept trial. The Lancet. |
|  | Trial registration: <https://ClinicalTrials.gov/show/NCT01184300> |
| **So, 2016**  ***(RAPID-STEMI; NCT01452139)*** | So DY, Wells GA, McPherson R, Labinaz M, Le May MR, Glover C, et al. A prospective randomized evaluation of a pharmacogenomic approach to antiplatelet therapy among patients with ST-elevation myocardial infarction: the RAPID STEMI study. Pharmacogenomics journal. 2016;16(1). |
|  | So DYF, Wells G, McPherson R, Labinaz M, Glover C, Le May M, et al. A pharmacogenomic approach to antiplatelet therapy in stemi patients: reassessment of anti-platelet therapy using an individualized strategy in patients with St-elevation myocardial infarction (the rapid STEMI study). Journal of the American College of Cardiology. 2013;61(10). |
|  | Trial registration: <https://ClinicalTrials.gov/show/NCT01452139> |
| **Koltowski, 2017**  ***(ONSIDE TEST;***  ***NCT01930773)*** | Koltowski L, Tomaniak M, Aradi D, Huczek Z, Filipiak KJ, Kochman J, et al. Optimal aNtiplatelet pharmacotherapy guided by bedSIDE genetic or functional TESTing in elective PCI patients: A pilot study: ONSIDE TEST pilot. Cardiology journal. 2017;24(3). |
|  | Tomaniak M, Koltowski L, Kochman J, Huczek Z, Rdzanek A, Pietrasik A, et al. Can prasugrel decrease the extent of periprocedural myocardial injury during elective percutaneous coronary intervention? Polish archives of internal medicine. 2017;127(11). |
|  | Tomaniak M, Koltowski L, Filipiak KJ, Kochman J, Huczek Z, Rdzanek A, et al. Can prasugrel decrease the extent of periprocedural myocardial injury during elective PCI?-first report from the ONSIDE TEST Study. European heart journal. 2017;38. |
|  | Koltowski L, Aradi D, Huczek Z, Tomaniak M, Sibbing D, Filipiak KJ, et al. Study design and rationale for Optimal aNtiplatelet pharmacotherapy guided by bedSIDE genetic or functional TESTing in elective percutaneous coronary intervention patients (ONSIDE TEST): a prospective, open-label, randomised parallel-group multicentre trial (NCT01930773). Kardiologia polska. 2016;74(4). |
|  | Trial registration: <https://ClinicalTrials.gov/show/NCT01930773> |
| **Voicu, 2024** | Voicu V, Diehm N, Moarof I, Parejo S, Badiqué F, Burden A, Niedrig D, Béchir M, Russmann S. Antiplatelet therapy guided by CYP2C19 point-of-care pharmacogenetics plus multidimensional treatment decisions. Pharmacogenomics. 2024 Jan;25(1):5-19. |
|  | Voicu V, Diehm N, Moarof I, Parejo S, Badique F, Burden A, et al. Personalized Antiplatelet Therapy based on Point-of-Care CYP2C19 Pharmacogenetics plus Multidimensional Treatment Decisions in a Cohort of 167 Patients. Swiss Medical Weekly. 2024;154:132S. |
| **Wirth, 2015** | Wirth F, Zahra G, Xuereb RG, Barbara C, Fenech A, Azzopardi LM. Comparison of a rapid point-of-care and two laboratory-based CYP2C19*2 genotyping assays for personalisation of antiplatelet therapy. International journal of clinical pharmacy. 2016;38(2). |
|  | Wirth F, Zahra G, Xuereb RG, Barbara C, Fenech A, Azzopardi LM. Comparison between a point-of-care and a laboratory-based CYP2C19* 2 genotyping assay for pharmacist-led personalisation of antiplatelet therapy. 2015. |
| **Zhou, 2017** | Zhou Y, Armstead AR, Coshatt GM, Limdi NA, Harada S. Comparison of Two Point-of-Care CYP2C19 Genotyping Assays for Genotype-Guided Antiplatelet Therapy. Annals of clinical and laboratory science. 2017;47(6). |
|  | Zhou Y, Armstead A, Coshatt G, Brott B, Sankaranarayanan A, Limdi N, et al., editors. Rapid CYP2C19 Genotype Testing: Comparison between Spartan RX CYP2C19 and Verigene CYP2C19. JOURNAL OF MOLECULAR DIAGNOSTICS; 2015: ELSEVIER SCIENCE INC 360 PARK AVE SOUTH, NEW YORK, NY 10010-1710 USA. |

## Excluded studies

The following table presents study reports excluded at full text with reasons for exclusion:

| **POCT** | **Citation** | **Reason for exclusion** |
| --- | --- | --- |
| Spartan FRX System | Spartan Bioscience, Yes, Children's Hospital of E, Ontario, Ottawa Hospital R, Institute, et al. 2012. Spartan FRX Project Reproducibility Study. NCT01676298; URL: <https://clinicaltrials.gov/show/NCT01676298> | Outcomes: No relevant outcomes measured or reported |
| Spartan FRX System | Spartan Bioscience I, Yes, Mount Sinai H, Canada Yes https clinicaltrials gov ct show results NCT, Nct. 2012. Method Comparison Study of the Spartan FRX CYP2C19 Genotyping System Against Bi-directional Sequencing. NCT01718535; URL: <https://clinicaltrials.gov/show/NCT01718535> | Duplicate study/report |
| Spartan RX System | Mayo C, Yes, Spartan B, Inc, Applied Health R, Centre, et al. 2012. Tailored Antiplatelet Therapy Following PCI. NCT01742117; URL: <https://clinicaltrials.gov/show/NCT01742117> | Duplicate study/report |
| Spartan RX System | Medical University of W, Yes, Polish C, Society, University o, Pecs, et al. 2013. Bedside Genetic or Pharmacodynamic Testing to Prevent Periprocedural Myonecrosis During PCI (ONSIDE TEST). NCT01930773; URL: <https://clinicaltrials.gov/show/NCT01930773> | Duplicate study/report |
| Spartan RX System | NCT01452139. 2011. Pharmacogenetic Approach to Anti-platelet Therapy for the Treatment of ST-segment Elevation Myocardial Infarction (STEMI). NCT01452139; URL: http://clinicaltrials.gov/show/NCT01452139). | Duplicate study/report |
| Spartan RX System | NCT01184300. 2010. ReAssessment of Anti-Platelet Therapy Using an InDividualized Strategy Based on GENetic Evaluation. URL: http://clinicaltrials.gov/show/NCT01184300). | Duplicate study/report |
| Spartan RX System | A Pharmacodynamic Study Comparing Prasugrel Versus Ticagrelor in Patients Undergoing PCI With CYP2C19 Loss-of-function:. 2014. NCT02065479; URL: <https://clinicaltrials.gov/show/NCT02065479> | Duplicate study/report |
| Spartan RX System | Assessment of Prospective CYP2C19 Genotype Guided Dosing of Anti-Platelet Therapy in Percutaneous Coronary Intervention. 2015. NCT02508116; URL: <https://clinicaltrials.gov/show/NCT02508116> | Duplicate study/report |
| Spartan RX System | Roberts JD, Wells GA, Le May MR, Labinaz M, Glover C, Froeschl M, et al. Point-of-care genetic testing for personalisation of antiplatelet treatment (RAPID GENE): a prospective, randomised, proof-of-concept trial. Lancet. 2012;379(9827) | Duplicate study/report |
| Spartan RX System | Spartan Cube CYP2C19 Inter Laboratory Reproducibilty Study. 2020. NCT04473573; URL: <https://clinicaltrials.gov/show/NCT04473573> | Outcomes: No outcome data we can report (it is confidential) |
| Spartan RX System | Spartan Cube CYP2C19 Method Comparison Study. 2020. NCT04473586; URL: <https://clinicaltrials.gov/show/NCT04473586> | Outcomes: No outcome data we can report (it is confidential) |
| Spartan RX System | Stimpfle F, Karathanos A, Droppa M, Metzger J, Rath D, Muller K, et al. Impact of point-of-care testing for CYP2C19 on platelet inhibition in patients with acute coronary syndrome and early dual antiplatelet therapy in the emergency setting. Thrombosis research 2014;134(1) | Outcomes: No relevant outcomes measured or reported |
| Spartan RX System | Tajima H, Izumi T, Miyachi S, Matsubara N, Ito M, Imai T, et al. Association between CYP2C19 genotype and the additional effect of cilostazol to clopidogrel resistance in neuroendovascular therapy. Nagoya journal of medical science 2018;80(2) | Outcomes: No relevant outcomes measured or reported |
| Spartan RX System | Tan SSN, Fong AYY, Mejin M, Gerunsin J, Kong KL, Chin FYY, et al. Association of CYP2C19*2 polymorphism with clopidogrel response and 1-year major adverse cardiovascular events in a multiethnic population with drug-eluting stents. Pharmacogenomics 2017;18(13) | Outcomes: No relevant outcomes measured or reported |
| Spartan RX System | University of F, Yes, National Institutes o, Health, National Human Genome R, Institute, et al. 2016. Implementation of CYP2C19 Genotyping to Guide Antiplatelet Therapy. NCT02724319; URL: <https://clinicaltrials.gov/show/NCT02724319> | Duplicate study/report |
| Spartan RX System | Vera, No HMD, Isala, Meander M, Center, Utrecht, et al. 2012. Cost-effectiveness of Genotype Guided Treatment With Antiplatelet Drugs in STEMI Patients: Optimization of Treatment (POPular Genetics). NCT01761786; URL: <https://clinicaltrials.gov/show/NCT01761786> | Duplicate study/report |
| Spartan RX System | Watanabe Y, Kozuma K, Ishikawa S, Hosogoe N, Isshiki T. Hyper-Response to Clopidogrel in Japanese Patients Undergoing Transcatheter Aortic Valve Implantation. International heart journal 2016;57(2) | Outcomes: No relevant outcomes measured or reported |
| Spartan RX System | Claassens DMF, van Dorst PWM, Vos GJA, Bergmeijer TO, Hermanides RS, van 't Hof AWJ, et al. Cost Effectiveness of a CYP2C19 Genotype-Guided Strategy in Patients with Acute Myocardial Infarction: results from the POPular Genetics Trial. American Journal of Cardiovascular Drugs 2021 | Duplicate study/report |
| Spartan RX System | Al-Rubaish AM, Al-Muhanna FA, Alshehri AM, Al-Mansori MA, Alali RA, Khalil RM, et al. Bedside testing of CYP2C19 vs. conventional clopidogrel treatment to guide antiplatelet therapy in ST-segment elevation myocardial infarction patients. International journal of cardiology. 2021;343. | Study aim is not to evaluate test |
| Spartan RX System | Al-Rubaish AM, Al-Muhanna FA, Alshehri AM, Al-Mansori MA, Alali RA, Khalil RM, et al. Bedside testing of CYP2C19 gene for treatment of patients with PCI with antiplatelet therapy. BMC cardiovascular disorders. 2020;20(1). | Study aim is not to evaluate test |
| Spartan RX System | Trial registration: https://ClinicalTrials.gov/show/NCT01823185 | Study aim is not to evaluate test |
| Spartan RX System | De Jin C, Kim MH, Guo LZ, Jin E, Shin E-S, Ann SH, et al. Pharmacodynamic study of prasugrel or clopidogrel in non-ST-elevation acute coronary syndrome with CYP2C19 genetic variants undergoing percutaneous coronary intervention (PRAISE-GENE trial). International Journal of Cardiology. 2020;305:11-7. | Study aim is not to evaluate test |
| Spartan RX System | Kim MH, Guo LZ, Shin ES, Ann SH, Cho YR, Park JS, et al. Relevance of the CYP2C19 polymorphism for loading and maintenance dose of prasugrel and clopidogrel treatment effect in coronary artery disease patients undergoing percutaneous coronary intervention: the PRAISE-GENE study. Journal of the American College of Cardiology. 2016;68(16). | Study aim is not to evaluate test |
| Spartan RX System | Kim SJ, Kim MH, Jin CD, Shin ES, Ann SH, Cho YR, et al. Prasugrel or clopidogrel in non-ST-elevation acute coronary syndrome with CYP2C19 genetic variants undergoing percutaneous coronary intervention. European heart journal. 2018;39. | Study aim is not to evaluate test |
| Spartan RX System | Trial registration: <https://clinicaltrials.gov/ct2/show/NCT01641510> | Study aim is not to evaluate test |
| Spartan RX System | Gross L, Trenk D, Jacobshagen C, Krieg A, Gawaz M, Massberg S, et al. Genotype-Phenotype Association and Impact on Outcomes following Guided De-Escalation of Anti-Platelet Treatment in Acute Coronary Syndrome Patients: the TROPICAL-ACS Genotyping Substudy. Thrombosis and haemostasis. 2018;118(9). | Study aim is not to evaluate test |
| Spartan RX System | Gross L, Kupka D, Trenk D, Geisler T, Hadamitzky M, Low A, et al. Gender and Outcomes following Guided De-Escalation of Antiplatelet Treatment in Acute Coronary Syndrome Patients: The TROPICAL-ACS Gender Substudy. Thrombosis and haemostasis. 2019;119(9). | Study aim is not to evaluate test |
| Spartan RX System | Sibbing D, Aradi D, Jacobshagen C, Gross L, Trenk D, Geisler T, et al. Guided de-escalation of antiplatelet treatment in patients with acute coronary syndrome undergoing percutaneous coronary intervention (TROPICAL-ACS): a randomised, open-label, multicentre trial. The Lancet. 2017;390(10104):1747-57. | Study aim is not to evaluate test |
| Spartan RX System | Sibbing D, Aradi D, Jacobshagen C, Gross L, Trenk D, Geisler T, et al. A randomised trial on platelet function-guided de-escalation of antiplatelet treatment in ACS patients undergoing PCI: Rationale and design of the Testing Responsiveness to Platelet Inhibition on Chronic Antiplatelet Treatment for Acute Coronary Syndromes (TROPICAL-ACS) Trial. Thrombosis and Haemostasis. 2017;117(1):188-95. | Study aim is not to evaluate test |
| Spartan RX System | Trial registration: <https://clinicaltrials.gov/ct2/show/NCT01959451> | Study aim is not to evaluate test |
| Spartan RX System | Kaikita K, Yoshimura H, Ishii M, Kudoh T, Yamada Y, Yamamoto E, et al. Tailored Adjunctive Cilostazol Therapy Based on CYP2C19 Genotyping in Patients With Acute Myocardial Infarction　- The CALDERA-GENE Study. Circulation journal. 2018;82(6). | Study aim is not to evaluate test |
| Spartan RX System | Trial registration: <https://center6.umin.ac.jp/cgi-open-bin/ctr_e/ctr_view.cgi?recptno=R000009423> | Study aim is not to evaluate test |
| Spartan RX System | Tuteja S, Glick H, Matthai W, Nachamkin I, Nathan A, Monono K, et al. Prospective CYP2C19 Genotyping to Guide Antiplatelet Therapy Following Percutaneous Coronary Intervention: A Pragmatic Randomized Clinical Trial. Circulation Genomic and precision medicine. 2020;13(1). | Study aim is not to evaluate test |
| Spartan RX System | Trial registration: <https://ClinicalTrials.gov/show/NCT02508116> | Study aim is not to evaluate test |
| Spartan RX System | Trial registration: <https://clinicaltrials.gov/ct2/show/NCT04619927> | Study aim is not to evaluate test |
| Genomadix Cube | Trial registration: <https://clinicaltrials.gov/ct2/show/NCT05773989> | Study aim is not to evaluate test |
| Multiplex PCR assay | Akkaif MA, Daud NAA, Noor DAM, Sha'aban A, Wahab Sk Abdul Kader MJA, Ibrahim B. Platelet reactivity index after treatment of clopidogrel versus ticagrelor based on CYP2C19 genotypes among patients undergoing percutaneous coronary intervention: results of a randomized study. European heart journal 2023;44 | Test: Not a POCT |
| NA | Kheiri B, Simpson TF, Osman M, Kumar K, Przybylowicz R, Merrill M, et al. Genotype-Guided Strategy for P2Y12 Inhibitors in Coronary Artery Disease: A Meta-Analysis of Randomized Clinical Trials. JACC: Cardiovascular Interventions 2020;13(5) | Publication: Not a primary study |
| None | Genotyping Guided Individualized Treatment of Clopidogrel and Ticagrelor in ACS. NCT02048228; URL: <https://ClinicalTrials.gov/show/NCT02048228> | Test: Not a POCT |
| None | Erlinge D, Gurbel PA, James S, Lindahl TL, Svensson P, Ten Berg JM, et al. Prasugrel 5 mg in the very elderly attenuates platelet inhibition but maintains noninferiority to prasugrel 10 mg in nonelderly patients: the GENERATIONS trial, a pharmacodynamic and pharmacokinetic study in stable coronary artery disease patients. Journal of the American College of Cardiology 2013;62(7) | Test: Not a POCT |
| None | Sanchez-Ramos J, Davila-Fajardo CL, Toledo Frias P, Diaz Villamarin X, Martinez-Gonzalez LJ, Martinez Huertas S, et al. Results of genotype-guided antiplatelet therapy in patients who undergone percutaneous coronary intervention with stent. International journal of cardiology 2016;225 | Test: Not a POCT |
| None | Suppiah V, Lim CX, Hotham E. Community pharmacists and their role in pharmacogenomics testing: An Australian perspective drawing on international evidence. Australian Journal of Primary Health 2018;24(6) | Publication: Not a primary study |
| Q3 System | Pharmacogenetics of Clopidogrel in Acute Coronary Syndromes. 2017. NCT03347435; URL: <https://clinicaltrials.gov/show/NCT03347435> | Test: Developed for research purposes only |
| Q3 System | Marziliano N, Notarangelo MF, Cereda M, Caporale V, Coppini L, Demola MA, et al. Rapid and portable, lab-on-chip, point-of-care genotyping for evaluating clopidogrel metabolism. Clinica Chimica Acta 2015;451 | Test: Developed for research purposes only |
| Q3 System | Pharmacogenetics of Clopidogrel in Acute Coronary Syndromes. NCT03347435; URL: https://ClinicalTrials.gov/show/NCT03347435. | Test: Developed for research purposes only |
| Q3 system | Notarangelo FM, Maglietta G, Bevilacqua P, Cereda M, Merlini PA, Villani GQ, et al. Pharmacogenomic Approach to Selecting Antiplatelet Therapy in Patients With Acute Coronary Syndromes: the PHARMCLO Trial. Journal of the American College of Cardiology 2018;71(17) | Test: Developed for research purposes only |
| Unclear | Vos GJA, Bergmeijer TO, Janssen PW, Asselbergs FW, Jhagroe D, Willemsen LW, et al. Tailored antiplatelet therapy based on CYP2C19 genotyping: a Popular Genetics pilot study. European heart journal 2016;37 | Test: Unclear; Outcomes: No relevant outcomes measured/ reported (no response from contacting authors) |
| Unclear | ZHU X, CHEN G, YAO M, SHI G, ZHOU X, ZHANG W, WANG L, LIU W, XU H, WANG C. Efficacy analysis of anti-platelet in the treatment of high-risk non-disabling ischemic cerebrovascular events guided by point-of-care testing of CYP2C19 gene. Chinese Journal of Neurology. 2023:365-73. | Test: Does not aim to evaluate POCT |
| Verigene System | Nct. 2010. Thrombocyte Activity Reassessment and GEnoTyping for PCI(TARGET-PCI). NCT01177592; URL: <https://clinicaltrials.gov/show/NCT01177592> | Test: POCT not used for CYP2C19 kit anymore; Study terminated |
| Verigene System | NCT01994941. 2013. Genotype Guided Versus Conventional Approach in Selection of Oral P2Y12 Receptor Blocker in Chinese Patients Suffering From Acute Coronary Syndrome. NCT01994941; URL: <https://clinicaltrials.gov/show/NCT01994941> | Test: POCT not used for CYP2C19 kit anymore |
| Verigene System | NCT01390974. 2011. Identification of Clopidogrel CYP2C19 Metabolizer and Thienopyridine Treatment After an Acute Coronary Syndrome. URL: http://clinicaltrials.gov/show/NCT01390974). | Test: POCT not used for CYP2C19 kit anymore |
| Verigene System | NCT01107925. 2010. Comparison of Prasugrel and Clopidogrel in Low Body Weight Versus Higher Body Weight With Coronary Artery Disease. URL: https://clinicaltrials.gov/show/NCT01107925). | Test: POCT not used for CYP2C19 kit anymore |
| Verigene System | NCT01107912. 2010. Comparison of Prasugrel and Clopidogrel in Very Elderly and Non-Elderly Patients With Stable Coronary Artery Disease. URL: https://clinicaltrials.gov/show/NCT01107912). | Test: POCT not used for CYP2C19 kit anymore |
| Verigene System | Ahn SG, Yoon J, Kim J, Uh Y, Kim KM, Lee JH, et al. Genotype- and phenotype-directed personalization of antiplatelet treatment in patients with non-ST elevation acute coronary syndromes undergoing coronary stenting. Korean circulation journal 2013;43(8) | Test: POCT not used for CYP2C19 kit anymore |
| Verigene System | Buchan BW, Peterson JF, Cogbill CH, Anderson DK, Ledford JS, White MN, et al. Evaluation of a microarray-based genotyping assay for the rapid detection of cytochrome P450 2C19 *2 and *3 polymorphisms from whole blood using nanoparticle probes. American journal of clinical pathology 2011;136(4) | Test: POCT not used for CYP2C19 kit anymore |
| Verigene System | Chae H, Kim M, Koh YS, Hwang BH, Kang MK, Kim Y, et al. Feasibility of a microarray-based point-of-care CYP2C19 genotyping test for predicting clopidogrel on-treatment platelet reactivity. BioMed research international 2013;2013 | Test: POCT not used for CYP2C19 kit anymore |
| Verigene System | P Collet JP, Kerneis M, Hulot JS, O'Connor SA, Silvain J, Mansencal N, et al. Point-of-care genetic profiling and/or platelet function testing in acute coronary syndrome. Thrombosis and haemostasis 2016;115(2) | Test: POCT not used for CYP2C19 kit anymore |
| Verigene System | Erlinge D, James S, Duvvuru S, Jakubowski JA, Wagner H, Varenhorst C, et al. Clopidogrel metaboliser status based on point-of-care CYP2C19 genetic testing in patients with coronary artery disease. Thrombosis and haemostasis 2014;111(5) | Test: POCT not used for CYP2C19 kit anymore |
| Verigene System | Kim H, Kim Y, Koh YS, Lee HK, Chae H, Jekarl DW, et al. Evaluation of the INNOVANCE PFA P2Y assay and its association with CYP2C19 genotypes. Platelets 2015;26(2) | Test: POCT not used for CYP2C19 kit anymore |
| Verigene System | Kong SL, Tam FCC, Wong AYT, Yung ASY, Shea PC, Chan EKY, et al. Efficacy of genotype guided selection of P2Y12 antagonist for treatment of acute coronary syndrome in Chinese patients. European heart journal 2016;37 | Test: POCT not used for CYP2C19 kit anymore |
| Verigene System | Nct. 2010. Thrombocyte Activity Reassessment and GEnoTyping for PCI(TARGET-PCI). NCT01177592; URL: https://clinicaltrials.gov/show/NCT01177592). | Duplicate study/report |
| Verigene System | NCT01994941. 2013. Genotype Guided Versus Conventional Approach in Selection of Oral P2Y12 Receptor Blocker in Chinese Patients Suffering From Acute Coronary Syndrome. NCT01994941; URL: https://clinicaltrials.gov/show/NCT01994941). | Duplicate study/report |
| Verigene System | Saracini C, Vestrini A, Galora S, Armillis A, Abbate R, Giusti B. Pharmacogenetics of clopidogrel: comparison between a standard and a rapid genetic testing. Genetic testing and molecular biomarkers 2012;16(6) | Test: POCT not used for CYP2C19 kit anymore |
| Verigene System | Tam CC, Kwok J, Wong A, Yung A, Shea C, Kong SL, et al. Genotyping-guided approach versus the conventional approach in selection of oral P2Y12 receptor blockers in Chinese patients suffering from acute coronary syndrome. The Journal of international medical research 2017;45(1) | Test: POCT not used for CYP2C19 kit anymore |
| Verigene System; AccuPower Real-time PCR CYP (*2,*3,*17) | Kim HK, Kang HJ, Ko DH, Jeong TD, Lee W, Chun S, et al. Comparison of the microarray-based assay, the real-time PCR assay, and the bidirectional sequencing method for CYP2C19 genotyping. Clinical laboratory 2015;61(8) | Test: POCT not used for CYP2C19 kit anymore |
| HyBeacon probes | Pirmohamed M, Burnside G, Eriksson N, Jorgensen AL, Toh CH, Nicholson T, et al. A randomized trial of genotype-guided dosing of warfarin. New England Journal of Medicine 2013;369(24) | Test: POCT does not measure CYP2C19 (different gene alleles and Hybeacon probes) also wrong drug (warfarin not clopidogrel)) |
| None | Biswas M. Global distribution of *CYP2C19* risk phenotypes affecting safety and effectiveness of medications. Pharmacogenomics. 2021;21(2). | Not a primary study: prevalence study (no data). |
| None | Chen X, Xu J, Chen S, Dong Q, Dong Y. Dual antiplatelet therapy with ticagrelor may increase the risk of all bleeding events in patients with minor strokes or high risk TIAs: a meta- analysis [published online ahead of print, 2022 Mar 3]. Stroke and vascular neurology*.* 2022; | Publication type: Meta-analysis |
| None | Dawson J, Merwick Á, Webb A, et al. European Stroke Organisation expedited recommendation for the use of short-term dual antiplatelet therapy early after minor stroke and high- risk TIA. European Stroke Journal. 2021;6(2). | Not a primary study: guideline. |
| None | Hao Q, Tampi M, O'Donnell M, Foroutan F, Siemieniuk RA, Guyatt G. Clopidogrel plus aspirin versus aspirin alone for acute minor ischaemic stroke or high risk transient ischaemic attack: systematic review and meta-analysis. British Medical Journal. 2018;363. | Publication type: Systematic review |
| None | Johnston SC, Amarenco P, Albers GW, Denison H, Easton JD, Evans SR, Held P, Jonasson J, Minematsu K, Molina CA, Wang Y, Wong KS; SOCRATES Steering Committee and Investigators. Ticagrelor versus Aspirin in Acute Stroke or Transient Ischemic Attack. New England Journal of Medicine. 2016 Jul 7;375(1). | Test: no reported *CYP2C19* genotyping |
| None | Lee CR, Luzum JA, Sangkuhl K, et al. Clinical Pharmacogenetics Implementation Consortium Guideline for *CYP2C19* Genotype and Clopidogrel Therapy: 2022 Update [published online ahead of print, 2022 Jan 16]. Clinical Pharmacology & Therapeutic. | Not a primary study: guideline. |
| None | Li C, Jia W, Li J, Li F, Ma J, Zhou L. Association with *CYP2C19* polymorphisms and Clopidogrel in treatment of elderly stroke patients. BMC Neurology*.* 2021;21(1):104. | Test: no reported *CYP2C19* genotyping |
| None | Li YJ, Chen X, Tao LN, Hu XY, Wang XL, Song YQ. Association between *CYP2C19* polymorphisms and clinical outcomes in patients undergoing stent procedure for cerebral artery stenosis. Scientific Reports . 2021;11(1). | Test: Study did not report an evaluation of POCT in scope |
| None | Luengo-Fernandez R, Violato M, Candio P, Leal J. Economic burden of stroke across Europe: A population-based cost analysis. European Stroke Journal. 2020;5(1). | Test/  Outcomes: no reported *CYP2C19* genotyping and study reports cost outcomes. |
| None | Anita Patel, Vladislav Berdunov, Derek King, Zahidul Quayyum, Raphael Wittenberg, Martin Knapp. *Current, future and avoidable costs of stroke in the UK*. London: Centre for Primary Care & Public Health, Queen Mary University of London, and the Personal Social Services Research Unit,; 2017. (Accessed November 2022)  <https://www.stroke.org.uk/sites/default/files/>  costs_of_stroke_in_the_uk_summary_report_0.pdf: | Not a primary study: report. |
| None | Pilling LC, Türkmen D, Fullalove H, et al. Analysis of *CYP2C19* genetic variants with ischaemic events in UK patients prescribed clopidogrel in primary care: a retrospective cohort study. *BMJ Open*. 2021;11(12). | Test: no reported *CYP2C19* genotyping |
| Spartan RX | Stimpfle F, Karathanos A, Droppa M, et al. Impact of point-of-care testing for *CYP2C19* on platelet inhibition in patients with acute coronary syndrome and early dual antiplatelet therapy in the emergency setting. Thrombosis Research. 2014;134(1) | Outcomes: no outcome data for Genomadix. |
| None | Uchino K. Guideline: Starting dual antiplatelet therapy ≤ 24 h after high-risk TIA or minor ischemic stroke is recommended. Annanls of Internal Medicine. 2019 Apr 16;170(8). | Not a primary study: guideline. |
| None | Wafa HA, Wolfe CDA, Emmett E, Roth GA, Johnson CO, Wang Y. Burden of Stroke in Europe: Thirty-Year Projections of Incidence, Prevalence, Deaths, and Disability-Adjusted Life Years. Stroke. 2020 Aug;51(8). | Not a primary study: modelling study. |

# Section 4: Data extraction and risk of bias assessments

## Accuracy of POCT for CYP2C19 LOF alleles

### Baseline Details

Note: All studies in the table below for objective 1, except for NCT01718535 (2) are also included for objective 2.

| **Study details** | **Participants*** | **POCT Test Details** | **Outcomes reported** |
| --- | --- | --- | --- |
| **Author, year:** Baudhuin et al (2022)(3, 4)  **Publication type:** Journal article  **Funding:** Non-industry  **Country:** US, Canada, South Korea, Mexico  **Start date:** NR  **Study name:** TAILOR-PCI  **Study design:** Diagnostic test accuracy cohort within an RCT | **Population:** Healthy people – pre-trial validation of test performance  **Inclusion/exclusion criteria:** NR  **Number of participants:** 373  **Mean age in years, SD, range:** NR  **Male %:** NR  **Ethnicity:** NR | **Test name:** Spartan RX (Genomadix Cube)  **Number of participants tested:** 373  **Alleles tested for:** *2, *3, *17  **Who administered test:** Onsite testing staff | Test accuracy  Ease of use of test |
|  | **Population:** Acute coronary syndrome or stable coronary artery disease and undergoing PCI – main trial  **Inclusion criteria:** 18+ years, target condition, planned 12 months of dual antiplatelet therapy (DAPT)  **Number of participants:** 2641  **Mean age in years, SD, range:** NR, NR, 26-95  **Male %:** 75  **Ethnicity:** 68% white, 23% east Asian, 4% south Asian, 2% African American, 2% other, 3% Hispanic or Latinx ethnicity | **Test name:** Spartan RX (Genomadix Cube)  **Number of participants tested:** 2587  **Alleles tested for:** *2, *3, *17  **Who administered test:** NR | Test accuracy  Test failure rate |
| **Author, year:** Choi et al. (2016)(5)  **Publication type:** Journal article  **Funding:** Non-industry  **Country:** South Korea  **Start date:** May 2013  **Study design:** Diagnostic test accuracy | **Population:** Acute coronary syndrome (ACS) undergoing PCI with drug-eluting stents  **Inclusion criteria:** Aged 18+, symptomatic ACS including unstable angina/ non-STEMI 12hr from onset, stenosis >70% on angiography  **Exclusion criteria:** Hemodynamic instability, malignancies, active bleeding, recent operation/ trauma, febrile disease, acute/ chronic inflammatory diseases, thrombocytopenia or anemia  **Number of participants:** 119  **Baseline data only reported by metaboliser status:**  **Mean age in years, SD:**  Poor: 62.5, 12.1; Intermediate: 61.9, 10.9; Extensive: 64.3, 13.6; Ultra-rapid: 64.8, 12.  **Male %:** Poor: 59.1%; Intermediate: 85.2%;  Extensive: 79.5%; Ultra-rapid: 75%.  **Ethnicity:** NR | **Test name:** Spartan RX (Genomadix Cube)  **Number of participants tested:** 119  **Alleles tested for:** *2, *3, *17  **Who administered test:** NR | Test accuracy  Time to results |
| **Author, year:** Genedrive.(2023)(6)  **Publication type:** Genedrive CYP2C19 ID kit  **Funding:** Industry – test manufacturer  **Country:** NR  **Start date:** NR  **Study design:** Diagnostic test accuracy | **Population:** Adult donor specimens  **Number of participants:** 250 donor specimens  **Mean age in years, SD, range:** NR  **Male %:** NR  **Ethnicity:** NR | **Test name:** Genedrive  **Number of participants tested:** 250  **Alleles tested for:** *1, *2, *3, *4, *8, *35, *17  **Who administered test:** NR | Test accuracy  Test failure rate  Time to results |
| **Author, year:** Meng et al.(2021)(7)  **Publication type:** Journal article  **Funding:** Non-industry  **Country:** China  **Start date:** July 2019  **Study design:** Diagnostic test accuracy cohort within the CHANCE-2 RCT | **Population:** Healthy individuals and patients with history of cardiovascular and cerebrovascular diseases from six hospitals  **Number of participants:** 408  **Mean age in years, SD, range:** 60.8, IQR 53.3-67.1, range 22-90 years  **Male %:** 66%  **Ethnicity:** Mainly Han Chinese patients | **Test name:** GMEX System  **Number of participants tested:** 408  **Alleles tested for:** *2, *3, *17  **Who administered test:** Doctors, nurses, clinical researchers | Test accuracy  Test failure rate  Time to results  Ease of use of test |
| **Author, year:** NCT01718535(2)  **Publication type:** Trial registration  **Funding:** Industry – test manufacturer  **Country:** Canada  **Start date:** September 2012  **Study design:** Diagnostic test accuracy | **Population:** NR  **Inclusion criteria:** Aged 16+  **Exclusion criteria:** None  **Number of participants:** 327  **Mean age in years, SD, range:** NR  **Male %:** NR  **Ethnicity:** NR | **Test name:** Spartan FRX (Genomadix Cube)  **Number of participants tested:** 325  **Alleles tested for:** *2, *3, *17  **Who administered test:** NR | Test accuracy |
| **Author, year:** Petrek et al.(2016)(8, 9)  **Publication type:** Journal Article  **Funding:** Unclear  **Country:** Czech Republic  **Start date:** March 2013  **Study design:** Diagnostic test accuracy | **Population:** PCI  **Inclusion criteria:** Random subset of patients  **Exclusion criteria:** NR  **Number of participants:** 53  **Mean age in years, range:** 57, 13-77  **Male %:** 74%  **Ethnicity:** NR | **Test name:** Spartan RX (Genomadix Cube)  **Number of participants tested:** 53  **Alleles tested for:** *2, *3, *17  **Who administered test:** NR | Test accuracy  Test failure rate  Time to results  Ease of use of test |
| **Author, year:** Roberts et al.(2012)(10)  **Publication type:** Journal article  **Funding:** Industry – test manufacturer  **Country:** Canada  **Start date:** 26 Aug 2010  **Study name:** RAPID GENE  **Study design:** RCT (diagnostic test accuracy cohort within an RCT) | **Population:** Undergoing PCI for treatment of non-ST-elevation ACS/ stable coronary artery disease.  **Inclusion criteria:** 18-75 years, followed-up >1 week  **Exclusion criteria:** Antiplatelet other than aspirin/ clopidogrel, or anticoagulation with warfarin/ dabigatran; history of stroke/ TIA; pregnancy; weight <60 kg; platelet <100 000 per μL; bleeding diathesis; haematocrit <30% or >52%, severe liver/renal disease  **Number of participants:** 200 (102 rapid genotyping arm; 98 standard arm genotyped later)  **Mean age in years, SD, range:** 60, 9, NR.  **Male %:** 80  **Ethnicity:** 95% white | **Test name:** Spartan RX (Genomadix Cube)  **Number of participants tested:** 200  **Alleles tested for:** *1, *2  **Who administered test:** Trial nurses | Test accuracy  Time to results  Ease of use of test |
| **Author, year:** So et al.(2016)(11)  **Publication type:** Journal article  **Funding:** Mixed (Industry – test manufacturer and non-industry)  **Country:** Canada  **Start date:** NR  **Study name:** RAPID-STEMI  **Study design:** Prospective randomized study (diagnostic test accuracy cohort within an RCT) | **Population:** PCI for STEMI.  **Inclusion criteria:** Aged 18-75; PCI for STEMI.  **Exclusion criteria:** Pre-treatment with prasugrel/ ticagrelor, need oral anti-coagulant, history of stroke/ TIA, body weight <60kg, platelet count <100,000 ul-1, bleeding diathesis, haemtocrit <30% or >52%, severe liver dysfunction, renal insufficiency, or <24hr treatment with glycoprotein IIb/IIIa inhibitors  **Number of participants:** 102  **Mean age in years, SD, range:** 58, 10, NR  **Male %:** 77  **Ethnicity:** 91% Caucasian | **Test name:** Spartan RX (Genomadix Cube)  **Number of participants tested:** 102  **Alleles tested for:** *2, *17  **Who administered test:** NR | Test accuracy  Time to results |
| **Author, year:** Voicu et al.(2024)(12, 13)  **Publication type:** Journal article  **Funding:** Not funded  **Country:** Switzerland  **Start date:** May 2022  **Study name:** NR  **Study design:** Diagnostic test accuracy | **Population:** Patients from specialities of cardiology, angiology and neurology with indication for clopidogrel genotyping  **Inclusion criteria:** As above  **Exclusion criteria:** NR  **Number of participants:** 167  **Median age in years, SD, range:** 70, NR, 34-91  **Male %:** 77.2  **Ethnicity:** NR | **Test name:** Genomadix Cube  **Number of participants tested:** 167  **Alleles tested for:** *2, *3, *17  **Who administered test:** Staff physicians collected sample, laboratory staff analysed the assay | Test accuracy  Time to results |
| **Author, year:** Wirth et al.(2016)(14, 15)  **Publication type:** Journal article  **Funding:** Industry – other  **Country:** Malta  **Start date:** October 2014  **Study design:** Diagnostic test accuracy | **Population:** PCI with stent for ACS/ stable angina; eligible for DAPT post-PCI  **Inclusion criteria:** As above  **Exclusion criteria:** Aged <18 or >75, weight <60 kg, history of stroke/ TIA, active bleeding, coagulation disorders, platelet disorders and/or chronic liver disease  **Number of participants:** 35  **Mean age in years, SD, range:** 65.8, 2.4, 49-75  **Male %:** 74  **Ethnicity:** 100% Caucasian | **Test name:** Spartan RX (Genomadix Cube)  **Number of participants tested:** 35  **Alleles tested for:** *2, *1  **Who administered test:** Clinical pharmacist researcher | Test accuracy  Test failure rate  Time to results  Ease of use of test  Cost of testing |

*When we are focusing on a cohort within an RCT, the ‘number of participants’ is the number of participants in the genotyping arm of a study (our cohort of interest), whilst the ‘total number of participants tested’ in the POCT column refers to the number tested with the POCT (not always the same number). Abbreviations: PCI: percutaneous coronary intervention, NR: not reported, NA: not applicable, SD: standard deviation, STEMI: ST-segment elevation myocardial infarction, RCT: randomised controlled trial, DAPT: dual antiplatelet therapy, ACS: acute coronary syndrome

### Results

| **Study details** | **Index test details (POCT)** | **Reference standard**  **(lab test)** | **Dataset** | **TP** | **FN** | **TN** | **FP** | **Sensitivity (95% CI)** | **Specificity (95% CI)** | **Discordant results** |
| --- | --- | --- | --- | --- | --- | --- | --- | --- | --- | --- |
| Baudhuin et al (2022)(3, 4) | **Test name:** Genomadix Cube/ Spartan  **Threshold for positive result:** *2 or *3 | **Test name**: CLIA-based *CYP2C19* Sanger sequencing  **Number participants tested:** 373  **Threshold for positive result:** NR | PRE-TRIAL | 151 | 0 | 224 | 0 | 100 | 100 | 2 discordant due to pre-analytical sample mix-up at testing centre. Samples re-collected and re-tested, then concordant. |
|  |  | **Test name**: Taqman  **Number participants tested:** 2385  **Threshold for positive result:** *2 or *3 | MAIN TRIAL* | 806 | 9 | 1561 | 9 | 99.0 | 99.0 | 21 discordant:  9 non-carrier by Spartan, but had *2 or *3 by TaqMan; 11 heterozygous *2 or *3 by Spartan, but non-carrier by TaqMan; 1 sample was heterozygous *2 by Spartan, but homozygous *2 by TaqMan. |
| Choi et al. (2016)(5) | **Test name:** Genomadix Cube/ Spartan  **Threshold for positive result:** *2, *3 | **Test name**: Taqman  **Number participants tested:** 119  **Threshold for positive result:** *2, *3 | NA | 76 | 0 | 43 | 0 | 100 | 100 | 2 discordant:- *3/*17 on Spartan and *1/*3 on SNP; *1/*17 on Spartan and *1/*1 on SNP |
| Genedrive.(2023)(6) | **Test name:** Genedrive  **Threshold for positive result:** *2, *3, *4, *8 or *35 | **Test name**: Buccal cell derived gDNA using the Agena MassARRAY and/or Taqman  **Number participants tested:** 250 specimens  **Threshold for positive result:** *2, *3, *4, *8 or *35 | NA | 83 | 0 | 167 | 0 | 100 (96, 100) | 100 (98, 100) | Four samples incorrectly classified by Genedrive – 2 samples with one LOF allele were classified as have 2 LOF alleles, and 2 samples with 2 LOF alleles were classified as having 1 LOF allele. |
| Meng et al.(2021)(7) | **Test name:** GMEX System  **Threshold for positive result:** *2 or *3 | **Test name**: Sanger sequencing  **Number participants tested:** 408  **Threshold for positive result:** *2 or *3 | NA | 242 | 0 | 166 | 0 | 100 | 100 | None |
|  | **Threshold for positive result:** *2 | **Test name:** Sanger sequencing  **Number participants tested:** 408  **Threshold for positive result:** *2 | NA | 217 | 0 | 191 | 0 | 100 | 100 | None |
|  | **Threshold for positive result:** *3 | **Test name:** Sanger sequencing  **Number participants tested:** 408  **Threshold for positive result:**  *3 | NA | 45 | 0 | 363 | 0 | 100 | 100 | None |
| NCT01718535.(2) | **Test name:** Genomadix Cube/ Spartan  **Threshold for positive result:** *2 or *3 | **Test name**: Bidirectional sequencing  **Number participants tested:** 325  **Threshold for positive result:** *2 or *3 | NA | 181 | 0 | 144 | 0 | 100 | 100 | None |
| Petrek et al. (2016)(8, 9) | **Test name:** Genomadix Cube/ Spartan  **Threshold for positive result:** *2, *3 | **Test name**: MassArray technology  **Number participants tested:** 53  **Threshold for positive result:** *2, *3 | NA | NR | NR | NR | NR | 100 | 100 | None |
| Roberts et al. (2012)(10) | **Test name:** Genomadix Cube/ Spartan  **Threshold for defining positive result:** *2 | **Test name**: DNA sequencing  **Number of participants tested:** 200 (data reported for 187 followed up)  **Threshold for defining positive result:** *2 | NA | 45 | 0 | 141 | 1 | 100% (95% CI 92.3-100) | 99.3% (95% CI 96.3-100) | One incorrectly classified as *2 carrier on Spartan |
| So et al. (2016)(11) | **Test name:** Genomadix Cube/ Spartan  **Threshold for positive result:** *2 | **Test name**: Taqman  **Number participants tested:** 102  **Threshold for positive result:** *2 | NA | 37 | 0 | 63 | 2 | 100% (95% CI 88.0-100) | 97% (88.5-99.5) | There were some FP but it was not clear how many or how these were discordant. |
| Voicu et al.(2024)(12, 13) | **Test name:** Genomadix Cube  **Threshold for positive result:** *2, *3 | **Test name**: MassARRAY MALDI-TOF system  **Number participants tested:** 167  **Threshold for positive result:** *2, *3 | NA | 54 | 0 | 113 | 0 | 100 | 100 | None |
| Wirth et al. (2016)(14, 15) | **Test name:** Genomadix Cube/ Spartan  **Threshold for positive result:** *2 | **Test name**: Taqman assay  **Number participants tested:** 35 (data for 34 due to inconclusive result)  **Threshold for positive result:** *2 | NA | 13 | 0 | 21 | 0 | 100 | 100 | One incorrectly classified as *2/*2 on Spartan vs one 2* on Taqman and on GenID |
|  |  | **Test name**: GenID assay  **Number participants tested:** 34  **Threshold for positive result:** *2 |  | 13 | 0 | 21 | 0 | 100 | 100 | None |

* Number of people with LOF alleles deduced from Table 2(3); it was not possible for numbers for both Taqman & Genomadix Cube to be correct in this table with the other information needed to calculate data for the 2x2 table; we therefore assumed that the numbers for Taqman were correct to allow us to construct our 2x2 table

Abbreviations: TP: true positive, FN: false negative, TN: true negative, FP: false positive, AUC ROC: area under the receiver operating characteristics curve, NR: not reported, NA: not applicable. Threshold for defining positive result: positive result meaning having loss of function.

### Risk of bias assessments

| **Study Details** | Baudhuin(2022)(3, 4)  *Pre-trial* |
| --- | --- |

| **Domain 1: Patient selection** | |
| --- | --- |
| 373 volunteer samples analysed- no information about condition etc. | |
| Was a consecutive or random sample of patients enrolled? | Unclear |
| Was a case-control design avoided? | Yes |
| Did the study avoid inappropriate exclusions? | Yes |
| Could the selection of patients have introduced bias? | **Low** |
| *Rationale for judgement:* Volunteer samples, no case control design and likely avoided innappropriate exclusions. | |

| **DOMAIN 2: INDEX TEST** | |
| --- | --- |
| Genomadix cube test - conducted on samples. Test conducted on-site by onsite testing staff. Suggests Genomadix test was conducted first, then the report was sent off to the lab along with a saliva sample for Sanger sequencing. | |
| Were the index test results interpreted without knowledge of the results of the reference standard? | Yes |
| Could the conduct or interpretation of the index test have introduced bias? | **Low** |
| *Rationale for judgement:* Test order means Genomadix cube results would be available before lab test | |

| **DOMAIN 3: REFERENCE STANDARD** | |
| --- | --- |
| Sanger sequencing by centralised laboratory - conducted after spartan test completed. | |
| Was an appropriate reference standard used | Yes |
| Were the reference results interpreted without knowledge of the results of the index test? | Unclear |
| Could the reference standard, its conduct, or its interpretation have introduced bias? | **Low** |
| *Rationale for judgement:* The reference standard is likely to correctly classify the target condition. It is unclear who interpreted the reference standard. The result is unlikely to have been influenced by knowledge of the results of the index test. | |

| **DOMAIN 4: FLOW AND TIMING** | |
| --- | --- |
| 373 samples tested and analysed | |
| Did all patients receive a reference standard? | Yes |
| Did patients receive the same reference standard? | Yes |
| Were all patients included in the analysis? | Yes |
| Could the selection of patients have introduced bias? | **Low** |
| *Rationale for judgement:* Patient flow was unlikely to have introduced bias - all patients received the same reference standard and were included in the analysis. | |

| OVERALL RISK OF BIAS | **LOW** |
| --- | --- |
| *Rationale for judgement*: No concerns | |

| **Study Details** | Baudhuin et al (2022)(3, 4)  *Main trial* |
| --- | --- |

| **Domain 1: Patient selection** | |
| --- | --- |
| Seems no inappropriate exclusions took place. | |
| Was a consecutive or random sample of patients enrolled? | Unclear |
| Was a case-control design avoided? | Yes |
| Did the study avoid inappropriate exclusions? | Yes |
| Could the selection of patients have introduced bias? | **Low** |
| *Rationale for judgement:* Unlikely that patient selection introduced bias as this is a subset of a randomised controlled trial, no case-control design and likely avoided inappropriate exclusions. | |

| **DOMAIN 2: INDEX TEST** | |
| --- | --- |
| Spartan Rx test. Test conducted on-site by onsite testing staff. Spartan test was conducted on patients, then Taqman conducted 12 months later. | |
| Were the index test results interpreted without knowledge of the results of the reference standard? | Unclear |
| Could the conduct or interpretation of the index test have introduced bias? | **Low** |
| *Rationale for judgement:* Blinding is unlikely to influence interpretation in this study, therefore it is at low risk of bias for this domain. | |

| **DOMAIN 3: REFERENCE STANDARD** | |
| --- | --- |
| Taqman assay conducted in the research laboratory. Spartan test was conducted on patients, then Taqman conducted 12 months later. | |
| Was an appropriate reference standard used | Yes |
| Were the reference results interpreted without knowledge of the results of the index test? | Unclear |
| Could the reference standard, its conduct, or its interpretation have introduced bias? | **Low** |
| *Rationale for judgement:* The reference standard is likely to correctly classify the target condition. It is unclear who interpreted the reference standard. The result is unlikely to have been influenced by knowledge of the results of the index test. | |

| **DOMAIN 4: FLOW AND TIMING** | |
| --- | --- |
| 2385 patients received both tests - this is our sample of interest.; NA | |
| Did all patients receive a reference standard? | Yes |
| Did patients receive the same reference standard? | Yes |
| Were all patients included in the analysis? | Yes |
| Could the selection of patients have introduced bias? | **Low** |
| *Rationale for judgement:* Patient flow was unlikely to have introduced bias - all patients received the same reference standard and were included in the analysis. | |

| OVERALL RISK OF BIAS | **LOW** |
| --- | --- |
| *Rationale for judgement:* No concerns | |

| **Study Details** | Choi et al. (2016)(5) |
| --- | --- |

| **Domain 1: Patient selection** | |
| --- | --- |
| Sampling procedure unclear. Not a case-control design. It seems the study avoided innapropriate exclusions. | |
| Was a consecutive or random sample of patients enrolled? | Unclear |
| Was a case-control design avoided? | Yes |
| Did the study avoid inappropriate exclusions? | Yes |
| Could the selection of patients have introduced bias? | **Low** |
| *Rationale for judgement:* There is not much information given about patient selection however it seems unlikely this will have introduced bias in the accuracy of the genetic test. A case-control design was avoided and it seems likely that the study avoided innapropriate exclusions. | |

| **DOMAIN 2: INDEX TEST** | |
| --- | --- |
| The index test is the Spartan RX *CYP2C19* and was conducted and interpreted by researchers. It aimed to identify the *2, *3 and *17 allele. Results determined by Spartan and confirmed by ref standard. | |
| Were the index test results interpreted without knowledge of the results of the reference standard? | Unclear |
| Could the conduct or interpretation of the index test have introduced bias? | **Low** |
| *Rationale for judgement:* Blinding is unlikely to influence interpretation in this study, therefore it is at low risk of bias for this domain. | |

| **DOMAIN 3: REFERENCE STANDARD** | |
| --- | --- |
| The reference standard was the Taqman SNP genotyping assay. It is unclear who conducted and interpreted it. Results determined by Spartan and confirmed by ref standard. | |
| Was an appropriate reference standard used | Yes |
| Were the reference results interpreted without knowledge of the results of the index test? | Unclear |
| Could the reference standard, its conduct, or its interpretation have introduced bias? | **Low** |
| *Rationale for judgement:* The reference standard is likely to correctly classify the target condition. It is unclear who interpreted and conducted the reference standard. The result is unlikely to have been influenced by knowledge of the results of the index test. | |

| **DOMAIN 4: FLOW AND TIMING** | |
| --- | --- |
| All patients received both tests.; NA | |
| Did all patients receive a reference standard? | Yes |
| Did patients receive the same reference standard? | Yes |
| Were all patients included in the analysis? | Yes |
| Could the selection of patients have introduced bias? | **Low** |
| *Rationale for judgement:* It seems unlikely that patient flow introduced bias- no missing data and all received same tests. | |

| OVERALL RISK OF BIAS | **Low** |
| --- | --- |
| *Rationale for judgement:* No concerns | |

| **Study Details** | Genedrive.(2023)(6) |
| --- | --- |

| **Domain 1: Patient selection** | |
| --- | --- |
| There is no information on study population/ patient selection. | |
| Was a consecutive or random sample of patients enrolled? | Unclear |
| Was a case-control design avoided? | Unclear |
| Did the study avoid inappropriate exclusions? | Unclear |
| Could the selection of patients have introduced bias? | Unclear |
| *Rationale for judgement:* There is no information on study population/ patient selection. | |

| **DOMAIN 2: INDEX TEST** | |
| --- | --- |
| The index test is the Genedrive test. There is no information about how it was conducted or interpreted. | |
| Were the index test results interpreted without knowledge of the results of the reference standard? | Unclear |
| Could the conduct or interpretation of the index test have introduced bias? | **Low** |
| *Rationale for judgement:* Blinding is unlikely to influence interpretation in this study, therefore it is at low risk of bias for this domain. | |

| **DOMAIN 3: REFERENCE STANDARD** | |
| --- | --- |
| The reference standard was the MassARRAY/ Taqman SNP genotyping assay. It is unclear who conducted and interpreted it. | |
| Was an appropriate reference standard used | Yes |
| Were the reference results interpreted without knowledge of the results of the index test? | Unclear |
| Could the reference standard, its conduct, or its interpretation have introduced bias? | **Low** |
| *Rationale for judgement:* The reference standard is likely to correctly classify the target condition. It is unclear who interpreted and conducted the reference standard. The result is unlikely to have been influenced by knowledge of the results of the index test. | |

| **DOMAIN 4: FLOW AND TIMING** | |
| --- | --- |
| All patients received both tests; NA | |
| Did all patients receive a reference standard? | Yes |
| Did patients receive the same reference standard? | Yes |
| Were all patients included in the analysis? | Yes |
| Could the selection of patients have introduced bias? | **Low** |
| *Rationale for judgement:* It seems unlikely that patient flow introduced bias. | |

| OVERALL RISK OF BIAS | **Unclear** |
| --- | --- |
| *Rationale for judgement:* Unclear due to limited information about population. | |

| **Study Details** | Meng et al.(2021)(7) |
| --- | --- |

| **Domain 1: Patient selection** | |
| --- | --- |
| 408 healthy individuals and patients with clinical history of cardiovascular and cerebrovascular diseases enrolled from six hospitals. Not clear if consecutive or random sample enrolled. | |
| Was a consecutive or random sample of patients enrolled? | Unclear |
| Was a case-control design avoided? | Yes |
| Did the study avoid inappropriate exclusions? | Yes |
| Could the selection of patients have introduced bias? | **Low** |
| *Rationale for judgement:* Healthy individuals/ patients, no case control design and likely avoided inappropriate exclusions. | |

| **DOMAIN 2: INDEX TEST** | |
| --- | --- |
| GMEX System conducted by trained doctors, nurses or clinical researchers. Not entirely clear but seems likely the GMEX System was conducted and interpreted first and then genotypes validated by Sanger sequencing. | |
| Were the index test results interpreted without knowledge of the results of the reference standard? | Yes |
| Could the conduct or interpretation of the index test have introduced bias? | **Low** |
| *Rationale for judgement:* Test order means GMEX System results would likely be available before lab test | |

| **DOMAIN 3: REFERENCE STANDARD** | |
| --- | --- |
| Sanger sequencing at the central laboratory – likely after GMEX System completed. | |
| Was an appropriate reference standard used | Yes |
| Were the reference results interpreted without knowledge of the results of the index test? | Unclear |
| Could the reference standard, its conduct, or its interpretation have introduced bias? | **Low** |
| *Rationale for judgement:* The reference standard is likely to correctly classify the target condition. It is unclear who interpreted the reference standard. The result is unlikely to have been influenced by knowledge of the results of the index test. | |

| **DOMAIN 4: FLOW AND TIMING** | |
| --- | --- |
| 408 samples tested and analysed. Samples for index test and reference standard taken at the same time. | |
| Did all patients receive a reference standard? | Yes |
| Did patients receive the same reference standard? | Yes |
| Were all patients included in the analysis? | Yes |
| Could the selection of patients have introduced bias? | **Low** |
| *Rationale for judgement:* Patient flow was unlikely to have introduced bias - all patients received the same reference standard and were included in the analysis. | |

| OVERALL RISK OF BIAS | **LOW** |
| --- | --- |
| *Rationale for judgement*: No concerns | |

| **Study Details** | NCT01718535.(2) |
| --- | --- |

| **Domain 1: Patient selection** | |
| --- | --- |
| "Recruitment of study participants was performed without knowledge of participant genotypes by enrolling associates of operators and associates of Spartan Bioscience and Mount Sinai Services", suggesting it was not consecutive or random. | |
| Was a consecutive or random sample of patients enrolled? | No |
| Was a case-control design avoided? | Yes |
| Did the study avoid inappropriate exclusions? | Yes |
| Could the selection of patients have introduced bias? | **Low** |
| *Rationale for judgement:* Patient selection was not random or consecutive, however the study wasn't limited to a specific condition, but it seems unlikely this would bias genetic test accuracy. A case-control design was avoided, and unlikely there were innappropriate exclusions. | |

| **DOMAIN 2: INDEX TEST** | |
| --- | --- |
| Spartan index test. No information about how tests were conducted and interpreted. Study states it is looking to identify *2, *3 and *17 allele. | |
| Were the index test results interpreted without knowledge of the results of the reference standard? | Unclear |
| Could the conduct or interpretation of the index test have introduced bias? | **Low** |
| *Rationale for judgement:* Blinding is unlikely to influence interpretation in this study, therefore it is at low risk of bias for this domain. | |

| **DOMAIN 3: REFERENCE STANDARD** | |
| --- | --- |
| Bidirectional sequencing is the lab test. No information about how it was conducted or interpreted. | |
| Was an appropriate reference standard used | Yes |
| Were the reference results interpreted without knowledge of the results of the index test? | Unclear |
| Could the reference standard, its conduct, or its interpretation have introduced bias? | **Low** |
| *Rationale for judgement:* The reference standard, bidirectional sequencing, is likely to correctly classify the target condition. The result is unlikely to have been influenced by knowledge of the results of the index test. | |

| **DOMAIN 4: FLOW AND TIMING** | |
| --- | --- |
| 327 patients enrolled but data analysed for 325. Two patients did not receive the reference standard (it says bidirectional sequencing not possible for 2 patients) - no reasoning provided for why this was.; NA | |
| Did all patients receive a reference standard? | No |
| Did patients receive the same reference standard? | Yes |
| Were all patients included in the analysis? | No |
| Could the selection of patients have introduced bias? | **Low** |
| *Rationale for judgement:* Missing data is low and all patients who received the reference standard received the same one. | |

| OVERALL RISK OF BIAS | **Low** |
| --- | --- |
| *Rationale for judgement:* No concerns | |

| **Study Details** | Petrek et al.(2016)(8, 9) | |
| --- | --- | --- |
|  | | |
| **Domain 1: Patient selection** | | |
| Methods of patient selection are not reported. All patients were undergoing acute coronary angioplasty with stent implantation for ACS. | | |
| Was a consecutive or random sample of patients enrolled? | | Unclear |
| Was a case-control design avoided? | | Yes |
| Did the study avoid inappropriate exclusions? | | Unclear |
| Could the selection of patients have introduced bias? | | **Low** |
| *Rationale for judgement:* There is not much information given about patient selection however it is unlikely this will have introduced bias in the accuracy of the genetic test. A case-control design was avoided. There is no information on exclusions but seems unlikely. | | |

| **DOMAIN 2: INDEX TEST** | |
| --- | --- |
| "Obtained samples were tested by Spartan RX AnalyserTM according to the operator’s manual". No information on how it was interpreted or order of tests. | |
| Were the index test results interpreted without knowledge of the results of the reference standard? | Unclear |
| Could the conduct or interpretation of the index test have introduced bias? | **Low** |
| *Rationale for judgement:* Blinding is unlikely to influence interpretation in this study, therefore it is at low risk of bias for this domain. | |

| **DOMAIN 3: REFERENCE STANDARD** | |
| --- | --- |
| The reference standard was MassArray technology. No information on how it was conducted and interpreted, other than to say "patients’ blood was sampled for DNA isolation and subsequent genotyping of *CYP2C19* polymorphisms" | |
| Was an appropriate reference standard used | Yes |
| Were the reference results interpreted without knowledge of the results of the index test? | Unclear |
| Could the reference standard, its conduct, or its interpretation have introduced bias? | **Low** |
| *Rationale for judgement:* The reference standard is likely to correctly classify the target condition. It is unclear who interpreted and conducted the reference standard.The result is unlikely to have been influenced by knowledge of the results of the index test. | |

| **DOMAIN 4: FLOW AND TIMING** | |
| --- | --- |
| All patients received the index test and reference standard and were included in the results.; NA | |
| Did all patients receive a reference standard? | Yes |
| Did patients receive the same reference standard? | Yes |
| Were all patients included in the analysis? | Yes |
| Could the selection of patients have introduced bias? | **Low** |
| *Rationale for judgement:* It seems unlikely that patient flow would have introduced bias - the tests were conducted simultaneously, all patients did receive the same reference standard and were included in the results. | |

| OVERALL RISK OF BIAS | **Low** |
| --- | --- |
| *Rationale for judgement:* No concerns | |

| **Study Details** | Roberts et al. (2012)(10) |
| --- | --- |

| **Domain 1: Patient selection** | |
| --- | --- |
| Patients who met the inclusion criteria were consecutively enrolled, then randomised. A case control design was avoided - all patients had the same condition. It seems the study avoided innappropriate exclusions. | |
| Was a consecutive or random sample of patients enrolled? | Yes |
| Was a case-control design avoided? | Yes |
| Did the study avoid inappropriate exclusions? | Yes |
| Could the selection of patients have introduced bias? | **Low** |
| *Rationale for judgement:* Low risk of bias because patients who met the inclusion criteria were consecutively enrolled, then randomised. | |

| **DOMAIN 2: INDEX TEST** | |
| --- | --- |
| The index test was Spartan RX *CYP2C19* point of care test. It was conducted by clinical trial nurses who had received a 30min training session but had no previous laboratory training. Seems Spartan test was conducted first and then the reference standard, but there is no information about interpretation of results. | |
| Were the index test results interpreted without knowledge of the results of the reference standard? | Unclear |
| Could the conduct or interpretation of the index test have introduced bias? | **Low** |
| *Rationale for judgement:* The conduct of the index test is outlined in the paper but the interpretation of the test is not. Blinding is unlikely to influence interpretation in this study, therefore it is at low risk of bias for this domain. | |

| **DOMAIN 3: REFERENCE STANDARD** | |
| --- | --- |
| The reference standard was DNA sequencing. DNA was extracted with the Arrow extaction robot and the Blood DNA 200 cartridge. Seems Spartan test was conducted first and then the reference standard, but there is no information about interpretation of results. | |
| Was an appropriate reference standard used | Yes |
| Were the reference results interpreted without knowledge of the results of the index test? | Unclear |
| Could the reference standard, its conduct, or its interpretation have introduced bias? | **Low** |
| *Rationale for judgement:* The reference standard is likely to correctly classify the target condition. It is unclear who interpreted and conducted the reference standard. The result is unlikely to have been influenced by knowledge of the results of the index test. | |

| **DOMAIN 4: FLOW AND TIMING** | |
| --- | --- |
| Test results reported for 91/102 randomised and tested in the genotyping arm, and 96/98 randomised and tested in the standard treatment arm. Missing patients were due to not undergoing PCI, being withdrawn by physician, undergoing different surgery, refusing to return for day 7 blood test and being lost to follow-up. | |
| Did all patients receive a reference standard? | Yes |
| Did patients receive the same reference standard? | Yes |
| Were all patients included in the analysis? | No |
| Could the selection of patients have introduced bias? | **Low** |
| *Rationale for judgement:* It seems unlikely that patient flow introduced bias. Not all patients are included in the analysis due to some being lost to follow-up but this doesn't seem like it is related to the true value. | |

| OVERALL RISK OF BIAS | **LOW** |
| --- | --- |
| Rationale for judgement: No concerns | |

| **Study Details** | So et al. (2016)(11) |
| --- | --- |

| **Domain 1: Patient selection** | |
| --- | --- |
| Prospectively enrolled patients meeting inclusion criteria from University of Ottawa Heart Institute - no further detail on sampling method. All patients had to have undergone PCI for STEMI. It seems there were no innappropriate exclusions. | |
| Was a consecutive or random sample of patients enrolled? | Unclear |
| Was a case-control design avoided? | Yes |
| Did the study avoid inappropriate exclusions? | Yes |
| Could the selection of patients have introduced bias? | **Low** |
| *Rationale for judgement:* Limited information on sampling technique but it seems unlikely this would bias the accuracy of the genetic test. A case control design was avoided. It seems the study avoided inappropriate exclusions. | |

| **DOMAIN 2: INDEX TEST** | |
| --- | --- |
| Spartan point of care test. Conducted appropriately, but no information on who did the test. Seems index test conducted/ interpreted first but limited explicit information on this. Threshold of looking for *2 allele specified. | |
| Were the index test results interpreted without knowledge of the results of the reference standard? | Unclear |
| Could the conduct or interpretation of the index test have introduced bias? | **Low** |
| *Rationale for judgement:* Blinding is unlikely to influence interpretation in this study, therefore it is at low risk of bias for this domain. | |

| **DOMAIN 3: REFERENCE STANDARD** | |
| --- | --- |
| Taqman assay. Conduct appropriate - extracting genomic DNA and underwent genetic analysis in the core laboratory. Seems index test conducted/ interpreted first but limited explicit information on this. | |
| Was an appropriate reference standard used | Yes |
| Were the reference results interpreted without knowledge of the results of the index test? | Unclear |
| Could the reference standard, its conduct, or its interpretation have introduced bias? | **Low** |
| *Rationale for judgement:* The reference standard is likely to correctly classify the target condition. It is unclear who interpreted and conducted the reference standard. The result is unlikely to have been influenced by knowledge of the results of the index test. | |

| **DOMAIN 4: FLOW AND TIMING** | |
| --- | --- |
| All patients received the tests and no exclusions.; NA | |
| Did all patients receive a reference standard? | Yes |
| Did patients receive the same reference standard? | Yes |
| Were all patients included in the analysis? | Yes |
| Could the selection of patients have introduced bias? | **Low** |
| *Rationale for judgement:* Patient flow was unlikely to have introduced bias. all patients received the same reference standard and were included in the analysis. | |

| OVERALL RISK OF BIAS | **Low** |
| --- | --- |
| Rationale for judgement: No concerns | |

| **Study Details** | Voicu et al.(2024)(12, 13) |
| --- | --- |

| **Domain 1: Patient selection** | |
| --- | --- |
| The study included all patients from specialities of cardiology, angiology and neurology with indication for CYP2C19 genotyping between May 2022 and June 2023. A case control design was avoided. | |
| Was a consecutive or random sample of patients enrolled? | Yes |
| Was a case-control design avoided? | Yes |
| Did the study avoid inappropriate exclusions? | Yes |
| Could the selection of patients have introduced bias? | **Low** |
| *Rationale for judgement:* The study used appropriate consecutive sampling and avoided a case control design and inappropriate exclusions. | |

| **DOMAIN 2: INDEX TEST** | |
| --- | --- |
| Genomadix cube conducted by staff physicians then analysed in the laboratory - not clear on order of interpretation but likely before ref standard. | |
| Were the index test results interpreted without knowledge of the results of the reference standard? | Unclear |
| Could the conduct or interpretation of the index test have introduced bias? | **Low** |
| *Rationale for judgement:* Blinding is unlikely to influence interpretation in this study, therefore it is at low risk of bias for this domain. | |

| **DOMAIN 3: REFERENCE STANDARD** | |
| --- | --- |
| Blood and buccal samples were both collected after the patients had signed informed consent. Blood samples were obtained by nursing staff and buccal swab samples by staff physicians, then sent to the laboratory for analysis. The reference standard was the MassARRAY MALDI-TOF system. Analyses performed by Labor Risch molecular genetics laboratory – order unclear. | |
| Was an appropriate reference standard used | Yes |
| Were the reference results interpreted without knowledge of the results of the index test? | Unclear |
| Could the reference standard, its conduct, or its interpretation have introduced bias? | **Low** |
| *Rationale for judgement:* The reference standard is likely to correctly classify the target condition. The result is unlikely to have been influenced by knowledge of the results of the index test. | |

| **DOMAIN 4: FLOW AND TIMING** | |
| --- | --- |
| All patients received all of the tests. No patients excluded from analysis. | |
| Did all patients receive a reference standard? | Yes |
| Did patients receive the same reference standard? | Yes |
| Were all patients included in the analysis? | Yes |
| Could the selection of patients have introduced bias? | **Low** |
| *Rationale for judgement:* It seems unlikely that patient flow introduced bias. | |

| OVERALL RISK OF BIAS | **LOW** |
| --- | --- |
| *Rationale for judgement*: No concerns | |

| **Study Details** | Wirth et al. (2016)(14, 15) |
| --- | --- |

| **Domain 1: Patient selection** | |
| --- | --- |
| The study used non-probability sampling. A case control design was avoided. | |
| Was a consecutive or random sample of patients enrolled? | No |
| Was a case-control design avoided? | Yes |
| Did the study avoid inappropriate exclusions? | Yes |
| Could the selection of patients have introduced bias? | **Low** |
| *Rationale for judgement:* The study used non-probability sampling but it seems unlikely this would bias the accuracy of the genetic test. | |

| **DOMAIN 2: INDEX TEST** | |
| --- | --- |
| Genomadix cube conducted and interpreted by a clinical pharmacist researcher before lab test - not clear on order of interpretation but likely before ref standard. | |
| Were the index test results interpreted without knowledge of the results of the reference standard? | Unclear |
| Could the conduct or interpretation of the index test have introduced bias? | **Low** |
| *Rationale for judgement:* Blinding is unlikely to influence interpretation in this study, therefore it is at low risk of bias for this domain. | |

| **DOMAIN 3: REFERENCE STANDARD** | |
| --- | --- |
| Both the taqman assay and the GenID assay were conducted by a clinical pharmacist researcher in liaison with a medical laboratory scientist at the Molecular Diagnostics Unit at Mater Dei Hospital MDH. They were classified by the clinical pharmacist researcher and classified in the same manner as with the Spartan RX assay. Seems ref standard interpreted and conducted after POCT. | |
| Was an appropriate reference standard used | Yes |
| Were the reference results interpreted without knowledge of the results of the index test? | Unclear |
| Could the reference standard, its conduct, or its interpretation have introduced bias? | **Low** |
| *Rationale for judgement:* The reference standard is likely to correctly classify the target condition. It is unclear who interpreted and conducted the reference standard. The result is unlikely to have been influenced by knowledge of the results of the index test. | |

| **DOMAIN 4: FLOW AND TIMING** | |
| --- | --- |
| All patients received all of the tests. One patient was excluded from the analysis as their Spartan index test was inconclusive and they could not be repeated as the patient had been discharged home.; NA | |
| Did all patients receive a reference standard? | Yes |
| Did patients receive the same reference standard? | Yes |
| Were all patients included in the analysis? | No |
| Could the selection of patients have introduced bias? | **Low** |
| *Rationale for judgement:* It seems unlikely that patient flow introduced bias. One patient was not included in results due to inconclusive result. | |

| OVERALL RISK OF BIAS | **LOW** |
| --- | --- |
| *Rationale for judgement*: No concerns | |

## Technical characteristics of POCT for CYP2C19 LOF alleles

Note: All studies included for objective 1, except for NCT01718535(2) are also included for objective 2. Studies that do not report accuracy data but reported technical characteristics data are listed in table below.

### Baseline Details

| **Study details** | **Participants*** | **POCT Test Details** | **Outcomes reported** |
| --- | --- | --- | --- |
| **Author, year:** Al-Rubaish et al. (2021)(16)  **Publication type:** Journal article  **Funding:** Non-industry  **Country:** Saudi Arabia  **Start date:** 2018  **Study design:** Technical performance study | **Population:** Ischaemic stroke  **Inclusion criteria:** Consecutive patients with ischaemic stroke  **Exclusion criteria:** NR  **Number of participants:** 256  **Mean age in years, SD, range:** 61, 12.5, 18-89  **Male %:** 65  **Ethnicity:** NR | **Test name:** Spartan RX (Genomadix Cube)  **Number of participants tested:** 256  **Alleles tested for:** *1, *2  **Who administered test:** NR | Time to results |
| **Author, year:** Azzahhafi et al.(2023)(17, 18)  **Publication type:** Journal article  **Funding:** Mixed – industry and non-industry  **Country:** the Netherlands  **Start date:** June 2021  **Study name:** NR  **Study design:** Technical performance study | **Population:** Acute coronary syndrome  **Inclusion criteria:** Patients in the FORCE-ACS Registry between June 2021-January 2023 who had been genotyped for *CYP2C19 and had complete data regarding their antiplatelet therapy.*  **Exclusion criteria:** NR  **Number of participants:** 855 (752 genotyped using POCT)  **Mean age in years, SD, range:** NR  **Male %:** NR  **Ethnicity:** NR | **Test name:** Genomadix (version unclear)  **Number of participants tested:** 752 (others tested with laboratory based system)  **Alleles tested for:** *2, *3, *17  **Who administered test:** Nurses | Time to results  Cost |
| **Author, year:** Bergmeijer et al.(2014)(19)  **Publication type:** Journal article  **Funding:** Non-industry (Spartan provided the tests)  **Country:** Netherlands, Italy, Belgium  **Study name:** The Popular Genetics Study  **Start date:** June 2011  **Study design:** Technical performance study | **Population:** ST-segment elevation myocardial infarction (STEMI)  **Inclusion criteria:** Aged ≥21; symptoms of acute myocardial infarction; primary PCI with stent implantation for STEMI  **Number of participants:** 1238  **Baseline data only provided for 1038/1238 participants as data not yet available for others**  **Mean age in years, SD, range:** 61.9, 11.2, NR  **Male %:** 74  **Ethnicity:** NR | **Test name:** Spartan RX (Genomadix Cube)  **Number of participants tested:** 411  **Alleles tested for:** *2, *3  **Who administered test:** Laboratory staff (1 site), local investigator or nurse (6 sites) | Test failure rate  Ease of use of test  Time to results |
| **Author, year:** Cavallari et al.(2018)(20)  **Publication type:** Journal article  **Funding:** Non-industry (Spartan provided genotyping platforms and kits)  **Country:** USA  **Start date:** April 28, 2016  **Study design:** Technical performance study | **Population:** Percutaneous coronary intervention (PCI)  **Inclusion criteria:** Patients undergoing emergent/ planned left heart catheterization with intent to undergo PCI  **Number of participants:** 931 patients genotyped (392 underwent PCI)  **Baseline data available only for those who underwent PCI:**  **Mean age in years, SD, range:** 63, 11, NR  **Male %:** 69  **Ethnicity:** White 74.5%, black 23.7%, asian 0.8%, other or not reported 1%. | **Test name:** Spartan RX (Genomadix Cube)  **Number of participants tested:** 931  **Alleles tested for:** *2, *3, *17  **Who administered test:** NR | Test failure rate  Time to results  Ease of use of test |
| **Author, year:** Davis et al. (2020)(21)  **Publication type:** Journal article  **Funding:** Non-industry.  **Country:** USA  **Start date:** NR  **Study design:** Diagnostic test accuracy study (but no relevant accuracy data for this review) | **Population:** NR  **Inclusion criteria:** NR  **Exclusion criteria:** NR  **Number of participants:** 23  **Age, sex, ethnicity:** NR | **Test name:** Spartan RX (Genomadix Cube)  **Number of participants tested:** 23  **Alleles tested for:** *2, *3, *17  **Who administered test:** NR | Ease of use of test |
| **Author, year:** Franchi et al.(2020)(22)  **Publication type:** Journal article  **Funding:** Non-industry (Spartan provided the Spartan RX system and reagents used free of charge)  **Country:** USA  **Start date:** NR  **Study design:** Technical performance study | **Population:** Diagnostic coronary angiography  **Inclusion criteria:** Consecutive patients aged 18-75 years scheduled to undergo diagnostic coronary angiography with intent to undergo ad hoc PCI  **Number of participants:** 781  **Age, sex, ethnicity:** NR | **Test name:** Spartan RX (Genomadix Cube)  **Number of participants tested:** 781  **Alleles tested for:** *1, *2, *3, *17  **Who administered test:** NR | Time to results |
| **Author, year:** Gurbel et al.(2024)(23-25)  **Publication type:** Journal article  **Funding:** NR  **Country:** USA  **Start date:** February 2017  **Study design:** Technical performance study | **Population:** Patients undergoing catheterisation  **Inclusion criteria:** NR  **Exclusion criteria:** NR  **Number of participants:** 1052 patients genotyped (429 underwent PCI)  **Baseline data available only for those who underwent PCI:**  **Mean age in years (SD):** 66 (12)  **Male %: 76**  **Ethnicity:** Caucasian 60% | **Test name:** Spartan RX (Genomadix Cube)  **Number of participants tested:** 1052  **Alleles tested for:** *1, *2, *3, *17  **Who administered test:** Trained staff | Time to results |
| **Author, year:** Levens et al.(2023)(26)  **Publication type:** Journal article  **Funding:** Not reported (Spartan provided genotyping system and kits)  **Country:** the Netherlands  **Start date:** September 2021  **Study name:** NR  **Study design:** Technical performance study | **Population:** Adult patients of 27 participating community pharmacies taking P2Y12 inhibitor-based antiplatelet therapy with either ticagrelor/ prasugrel, not previously *CYP2C19* genotyped.  **Inclusion criteria:** As above  **Exclusion criteria:** Contraindication for clopidogrel; COVID-19 or other respiratory disease infection during recruitment  **Number of participants:** 144  **Mean age in years, SD, range:** 64, NR, 34-87  **Male %:** 77  **Ethnicity:** Caucasian 97% | **Test name:** Genomadix (version unclear)  **Number of participants tested:** 144  **Alleles tested for:** *2, *3, *17  **Who administered test:** Patients collected own buccal swab (due to COVID-19 pandemic). If self-collection not possible, pharmacy staff collected swab. | Time to results  Test failure rate  Ease of use  Cost |
| **Author, year:** McDermott et al.(2020)(16, 27)  **Conference poster/ abstract**  **Funding:** NR  **Country:** United Kingdom  **Start date:** NR  **Study design:** Technical performance study | **Population:** NR  **Inclusion criteria:** NR  **Exclusion criteria:** NR  **Number of participants:** NR  **Age, sex, ethnicity:** NR | **Test name:** Genedrive (early version)  **Number of participants tested:** NR  **Alleles tested for:** *1,*2,*3,*4,*4b,*10, *17  **Who administered test:** NR | Time to results  Ease of use of test |
| **Author, year:** Koltowski et al.(2017)(28)  **Publication type:** Journal article  **Funding:** Non-industry  **Country:** Poland  **Start date:** NR  **Study name:** ONSIDE TEST study  **Study design:** Technical performance study | **Population:** Patients scheduled for elective PCI with stent implantation  **Number of participants:** 16 (genotyped)  **Mean age in years, SD, range:** 61.2, 10.2  **Male %:** 56%  **Ethnicity:** NR | **Test name:** Spartan RX (Genomadix Cube)  **Number of participants tested:** 16  **Alleles tested for:** *1, *2  **Who administered test:** Nurses or physicians | Test failure rate  Time to results  Ease of use of test |

| **Study details** | **Participants*** | **POCT Test Details** | **Outcomes reported** |
| --- | --- | --- | --- |
| **Author, year:** Zhou et al. (2017)(29, 30)  **Publication type:** Journal article  **Funding:** Non-industry  **Country:** USA  **Start date:** NR  **Study design:** Diagnostic test accuracy (but no accuracy data relevant for this review) | **Population:** Volunteers and control samples – condition NR - for validation of the test  **Number of participants:** 12 samples (9 volunteers, 3 Coriell samples, 4 CAP survey samples)  **Age, sex, ethnicity:** NR | **Test name:** Spartan RX (Genomadix Cube)  **Number of participants tested:** 12 samples  **Alleles tested for:** *2, *3, *17  **Who administered test:** Four laboratory technologists | Time to results |
|  | **Population:** Post-PCI patients  **Number of participants:** 342  **Age, sex, ethnicity:** NR | **Test name:** Spartan RX (Genomadix Cube)  **Number of participants tested:** 342  **Alleles tested for:** *2, *3, *17  **Who administered test:** NR | Test failure rate  Time to results |

### Results

| **Study details** | **Test name** | **Alleles tested for** | **Outcomes** | **Results** |
| --- | --- | --- | --- | --- |
| Al-Rubaish et al.(2021)(16) | Spartan RX (Genomadix Cube) | *1, *2 | Time to results | First 50 patients: 90-120min to complete the results |
| Azzahhafi et al.(2023)(17) | Genomadix (version unclear) | *2, *3, *17 | Time to results | Median turnover time with POCT was 5.7hr (IQR 2.0-12.6), with 90.5% of test results known within 24hr and 96.9% within 48hr. |
|  |  |  | Cost | The cost per POCT genotyping analysis (sum of disposable and employee costs) was 150 euros, while the cost per laboratory-based test was 75 euros (sum of machine and employee costs. |
| Baudhuin et al.(2022)(3, 4)  **Pre-trial** | Spartan RX (Genomadix Cube) | *2, *3, *17 | Ease of use of test | Non laboratory trained personnel can successfully perform rapid genotyping in a POC setting |
| Baudhuin et al.(2022)(3, 4)  **Main trial** | Spartan RX (Genomadix Cube) | *2, *3, *17 | Test failure rate | 172 (6%) patients with unavailable test result. 54/2642 (2%) had no Spartan result available (no definition of what this means); 118 (4%) had inconclusive results. |
| Bergmeijer et al.(2014)(19) | Spartan RX (Genomadix Cube) | *2, *3 | Test failure rate | 39 (8%) patients with unavailable test result - inconclusive results. |
|  |  |  | Ease of use of test | **Description of feature of the test:** Buccal swab more patient friendly than venapuncture for blood sample, but test is limited to testing *2, *3, *17 for one patient at a time per genotyping device. |
|  |  |  | Time to results | Result available within 1hr after collection of buccal swab. |
| Cavallari et al.(2018)(20) | Spartan RX (Genomadix Cube) | *2, *3, *17 | Test failure rate | 129 (14%) with unavailable test result - 56 inconclusive results and 73 device errors. |
|  |  |  | Time to results | For all patients genotyped: Median genotype test turnaround time was 96min (interquartile range of 78-144) |
|  |  |  | Ease of use of test | Could not be used as POCT due to absence of licensed molecular medical technologist so must be sent to central laboratory (the case for all of USA), and only a single sample genotyped at a time limiting number of patients that can be offered genotyping. |
| Choi et al.(2016)(5) | Spartan RX (Genomadix Cube) | *2, *3, *17 | Time to results | **Description of feature of the test:** time from sample to result ~60min |
| Davis et al.(2020)(21) | Spartan RX (Genomadix Cube) | *2, *3, *17 | Ease of use of test | **Description of features of the test**: Barriers to implementation: time constraints, personnel requirements and coordination, storage and sample stability, samples unable to be collected by bedside nurses, patients unable to provide samples, sample recollection due to interference or improper techniques |
| Franchi et al.(2020)(22) | Spartan RX (Genomadix Cube) | *1, *2, *3, *17 | Time to results | Allele status within 1hr - readily available when the decision on choice of oral P2Y12-inhibiting therapy most commonly occurs. |
| Gurbel et al.(2024)(23) | Spartan RX (Genomadix Cube) | *1, *2, *3, *17 | Time to results | Results available within 1 hour and the results were available immediately for the decision making for the cardiologist. |
| Levens et al.(2023)(26) | Genomadix test (version unclear) | *2, *3, *17 | Time to results | The average turnaround time was approximately 75 min. This time included pre-test counselling, buccal swap sample collection, hands-on genotyping, and 1 h runtime of the CYP2C19 POC device. |
|  |  |  | Test failure rate | Five patients (3.5%) had “erroneous test results” at first attempt. Of these five patients: two were excluded from the study (one withdrew, the other had two consecutive inconclusive results), two had a successful test result on the second attempt, and one had a successful test result on the third attempt. |
|  |  |  | Ease of use | **Patients:** 119 patients participated in a telephone survey. Almost all were positive about the convenience of testing in the community pharmacy and felt it was a suitable location for testing – 61% said they would rather undergo testing in the pharmacy than in the laboratory or hospital (mainly due to distance to pharmacy and shorter time to testing). Most patients felt the testing was easy, had increased understanding of the testing and saw the benefits of it when using medication. Patients appeared to feel the testing added value to health care, they had confidence in the pharmacist in conducting and interpreting the test and felt communication was clear. Overall, patients were positive about POCT in community pharmacy.  **Pharmacists:** Fourteen pharmacists participated in a telephone questionnaire. The most common reported barriers to testing with POCT were poor accessibility and non-cooperation of treating cardiologists, subpar knowledge and education of pharmacists, and testing being a difficult subject for patients. When asked to rank ten well-known perceived barriers to POCT testing, the highest ranked barriers were a lack of reimbursement and costs associated with testing, followed by limited time to commit to testing and insufficient staff. Reported facilitators included no resistance from patients, the added value of testing and that it is the future.  **Cardiologists:** Eight cardiologists participated in an online survey. Almost all felt the testing added value but three indicated they needed more assistance to put it into practice. Barriers to implementation included insufficient knowledge about testing, unsure if results will influence prescribing policy, increased administrative burden if results not immediately in patient record, and the time burden. Facilitators included personalised medicine being beneficial and the willingness to reduce healthcare costs due to use of ticagrelor and prasugrel. |
|  |  |  | Cost | 140 euros for the test materials, plus 10 euros for pharmacist time. |
| Meng et al.(2021)(7) | GMEX System | *2, *3, *17 | Test failure rate | None of the patients were retested or excluded owing to incorrect operation or first-run test failure in the GMEX genetic testing group. |
|  |  |  | Time to results | Average workflow length was 85.0min (IQR: 85.0–86.0). Results available ~1.5hr after sample collection (faster than laboratory-based genotyping (2–3) days). The time of sample-to-start, start-to-end and end-to-reports were 6.0 (IQR: 5.0–6.0), 62.0 (IQR: 61.5–62.0) and 18.0 (IQR: 18.0–18.0) min, respectively. |
|  |  |  | Ease of use of test | **Description of features of the test:** User-friendly and can be easily operated, transmitted and analysed and presents an effective and simple approach to genotyping. It is in line with the affordable, sensitive, specific, user-friendly, rapid and robust, equipment-free and deliverable to end-users criteria proposed by the WHO. |
| Petrek et al.(2016)(8, 16) | Spartan RX (Genomadix Cube) | *2, *3, *17 | Test failure rate | 10 (18.9%) with unavailable test result due to failure during amplification process (n=4), inconclusive result (n=3), only two of three alleles tested for gave results (n=3) |
|  |  |  | Time to results | Turnaround time (from buccal swab sampling to result print-out) was 60 min |
|  |  |  | Ease of use of test | Simple and non-invasive |
| Roberts et al.(2012)(10) | Spartan RX (Genomadix Cube) | *1, *2 | Time to results | Within 60min from test activation |
|  |  |  | Ease of use of test | Nurses with no previous laboratory training implemented test after 30min training session. |
| So et al.(2016)(11) | Spartan RX (Genomadix Cube) | *2, *17 | Time to results | Within 55min of test carrier status for all alleles was available |
| Koltowski et al.(2017)(28) | Spartan RX (Genomadix Cube) | *1, *2 | Test failure rate | Test had to be repeated for 2 patients due to inconclusive results |
|  |  |  | Time to results | Genotyping took 60min |
|  |  |  | Ease of use of test | Confirmed the feasibility and convenience of point-of-care testing – could be performed by physician or nurse subsequent to short training and results achieved quickly. |
| Voicu et al.(2024)(12, 13) | Genomadix Cube | *2, *3, *17 | Time to results | 117 (70.1%) of the Genomadix Cube results were available for treatment decisions within 24hr, and the remaining 50 (29.9%) were available within one week. |
| Wirth et al.(2016)(14, 16) | Spartan RX (Genomadix Cube) | *2, *1 | Test failure rate | 5/35 (14.3%) patients with unavailable test result – 4 tests resulted in error (11.4% - no further details); 1 test inconclusive. |
|  |  |  | Time to results | Collection of sample to genotyping result within 1 hour |
|  |  |  | Ease of use of test | Simple procedure, portable, convenient, no laborious preparation, minimal training required to conduct test. User-friendly interpretation with no training required. Storage conditions limit ease of use. |
|  |  |  | Cost of testing | Estimated cost per patient test: 225 euros (Taqman estimated at 13 euros and GenID at 23 euros). No indication of how this was calculated. |
| Zhou et al.(2017)(29, 30)  **Pre trial** | Spartan RX (Genomadix Cube) | *2, *3, *17 | Time to results | **Description of feature of the test (pre trial and main trial):** results are returned in one hour turnaround time |
| Zhou et al.(2017) (29, 30)  **Main trial** | Spartan RX (Genomadix Cube) | *2, *3, *17 | Test failure rate | 25 (7.3%) with unavailable test results - 14 inconclusive results (4%), 10 failed controls (3%), 1 instrument failure (0.3%) (no further information given). |
|  |  |  | Time to results | **Description of feature of the test (pre trial and main trial):** results are returned in one hour turnaround time |
| Genedrive.(2023)(6) | Genedrive | *1, *2, *3, *4, *8, *35, *17 | Time to results | The time from specimen collection to result was reported as 69 minutes plus an additional 3 minutes assay set up time |
|  |  |  | Test failure rate | Test failure rate was reported at 0.6% (358/360), but it was unclear whether the two test failures were based on donor or contrived samples and whether this was for the initial run only or after re-testing of failed samples |
| McDermott et al.(2020)(27, 31) | Genedrive | *1, *2, *3, *4, *8, *35, *17 | Time to results | **Description of feature of the test:** ~40min |
|  |  |  | Ease of use of test | **Description of features of the test**: Portable, rapid (~40mins), no cold chain, simple read out for non-specialist users. |

# Section 5: PRISMA Reporting checklist

| **Section/topic** | **#** | **PRISMA-DTA Checklist Item** | **Reported on page #** |
| --- | --- | --- | --- |
| **TITLE / ABSTRACT** | | |  |
| Title | 1 | Identify the report as a systematic review (+/- meta-analysis) of diagnostic test accuracy (DTA) studies. | 0 |
| Abstract | 2 | Abstract: See PRISMA-DTA for abstracts. | 1 |
| **INTRODUCTION** | | |  |
| Rationale | 3 | Describe the rationale for the review in the context of what is already known. | 2 |
| Clinical role of index test | D1 | State the scientific and clinical background, including the intended use and clinical role of the index test, and if applicable, the rationale for minimally acceptable test accuracy (or minimum difference in accuracy for comparative design). | 2 |
| Objectives | 4 | Provide an explicit statement of question(s) being addressed in terms of participants, index test(s), and target condition(s). | 3 |
| **METHODS** | | |  |
| Protocol and registration | 5 | Indicate if a review protocol exists, if and where it can be accessed (e.g., Web address), and, if available, provide registration information including registration number. | 3 |
| Eligibility criteria | 6 | Specify study characteristics (participants, setting, index test(s), reference standard(s), target condition(s), and study design) and report characteristics (e.g., years considered, language, publication status) used as criteria for eligibility, giving rationale. | 3 |
| Information sources | 7 | Describe all information sources (e.g., databases with dates of coverage, contact with study authors to identify additional studies) in the search and date last searched. | 3 |
| Search | 8 | Present full search strategies for all electronic databases and other sources searched, including any limits used, such that they could be repeated. | 3 + supp material section 2 |
| Study selection | 9 | State the process for selecting studies (i.e., screening, eligibility, included in systematic review, and, if applicable, included in the meta-analysis). | 4 |
| Data collection process | 10 | Describe method of data extraction from reports (e.g., piloted forms, independently, in duplicate) and any processes for obtaining and confirming data from investigators. | 4 |
| Definitions for data extraction | 11 | Provide definitions used in data extraction and classifications of target condition(s), index test(s), reference standard(s) and other characteristics (e.g. study design, clinical setting). | 4 |
| Risk of bias and applicability | 12 | Describe methods used for assessing risk of bias in individual studies and concerns regarding the applicability to the review question. | 4 |
| Diagnostic accuracy measures | 13 | State the principal diagnostic accuracy measure(s) reported (e.g. sensitivity, specificity) and state the unit of assessment (e.g. per-patient, per-lesion). | 4 |
| Synthesis of results | 14 | Describe methods of handling data, combining results of studies and describing variability between studies. This could include, but is not limited to: a) handling of multiple definitions of target condition. b) handling of multiple thresholds of test positivity, c) handling multiple index test readers, d) handling of indeterminate test results, e) grouping and comparing tests, f) handling of different reference standards | 4 |

Page 1 of 2

| **Section/topic** | **#** | **PRISMA-DTA Checklist Item** | **Reported on page #** |
| --- | --- | --- | --- |
| Meta-analysis | D2 | Report the statistical methods used for meta-analyses, if performed. | 4 |
| Additional analyses | 16 | Describe methods of additional analyses (e.g., sensitivity or subgroup analyses, meta-regression), if done, indicating which were pre-specified. | NA |
| **RESULTS** | | |  |
| Study selection | 17 | Provide numbers of studies screened, assessed for eligibility, included in the review (and included in meta-analysis, if applicable) with reasons for exclusions at each stage, ideally with a flow diagram. | 5 |
| Study characteristics | 18 | For each included study provide citations and present key characteristics including: a) participant characteristics (presentation, prior testing), b) clinical setting, c) study design, d) target condition definition, e) index test, f) reference standard, g) sample size, h) funding sources | Results section/ supp material section 4 |
| Risk of bias and applicability | 19 | Present evaluation of risk of bias and concerns regarding applicability for each study. | 6 |
| Results of individual studies | 20 | For each analysis in each study (e.g. unique combination of index test, reference standard, and positivity threshold) report 2x2 data (TP, FP, FN, TN) with estimates of diagnostic accuracy and confidence intervals, ideally with a forest or receiver operator characteristic (ROC) plot. | Results section/ supp material section 4 |
| Synthesis of results | 21 | Describe test accuracy, including variability; if meta-analysis was done, include results and confidence intervals. | Results section |
| Additional analysis | 23 | Give results of additional analyses, if done (e.g., sensitivity or subgroup analyses, meta-regression; analysis of index test: failure rates, proportion of inconclusive results, adverse events). | NA (but test failure covered under obj 2) |
| **DISCUSSION** | | |  |
| Summary of evidence | 24 | Summarize the main findings including the strength of evidence. | 9-11 |
| Limitations | 25 | Discuss limitations from included studies (e.g. risk of bias and concerns regarding applicability) and from the review process (e.g. incomplete retrieval of identified research). | 9-11 |
| Conclusions | 26 | Provide a general interpretation of the results in the context of other evidence. Discuss implications for future research and clinical practice (e.g. the intended use and clinical role of the index test). | 9-11 |
| **FUNDING** | | |  |
| Funding | 27 | For the systematic review, describe the sources of funding and other support and the role of the funders. | 12 |

*Adapted From:*  McInnes MDF, Moher D, Thombs BD, McGrath TA, Bossuyt PM, The PRISMA-DTA Group (2018). Preferred Reporting Items for a Systematic Review and Meta-analysis of Diagnostic Test Accuracy Studies: The PRISMA-DTA Statement. JAMA. 2018 Jan 23;319(4):388-396. doi: 10.1001/jama.2017.19163.

# References

1. Wang A, Tian X, Xie X, Li H, Bath PM, Jing J, et al. Differential effect of ticagrelor versus clopidogrel by homocysteine levels on risk of recurrent stroke: a post hoc analysis of the CHANCE-2 trial. CMAJ : Canadian Medical Association journal. 2024;196(5):E149‐E56.

2. Method Comparison Study of the Spartan FRX CYP2C19 Genotyping System Against Bi-directional Sequencing.

3. Baudhuin LM, Train LJ, Goodman SG, Lane GE, Lennon RJ, Mathew V, et al. Point of care CYP2C19 genotyping after percutaneous coronary intervention. Pharmacogenomics journal. 2022;22(5).

4. Baudhuin L, Train L, Goodman S, Lane G, Lennon R, Mathew V, et al. VALIDATION AND PERFORMANCE OF POINT-OF-CARE RAPID CYP2C19 GENOTYPING IN THE TAILOR-PCI MULTICENTER INTERNATIONAL RANDOMIZED CLINICAL TRIAL. Journal of the American College of Cardiology. 2021;77(18).

5. Choi J-L, Kim B-R, Woo K-S, Kim K-H, Kim J-M, Kim M-H, et al. The Diagnostic Utility of the Point-of-Care CYP2C19 Genotyping Assay in Patients with Acute Coronary Syndrome Dosing Clopidogrel: Comparison with Platelet Function Test and SNP Genotyping. Annals of clinical and laboratory science. 2016;46(5).

6. Genedrive. CYP2C19 ID Kit. 2023.

7. Meng X, Wang A, Zhang G, Niu S, Li W, Han S, et al. Analytical validation of GMEX rapid point-of-care CYP2C19 genotyping system for the CHANCE-2 trial. Stroke and vascular neurology. 2021;6(2).

8. Petrek M, Kocourkova L, Zizkova V, Nosek Z, Taborsky M, Petrkova J. Characterization of Three CYP2C19 Gene Variants by MassARRAY and Point of Care Techniques: Experience from a Czech Centre. Archivum immunologiae et therapiae experimentalis. 2016;64.

9. Petrkova J, Paskova L, Zizkova V, Nosek Z, Taborsky M, Petrek M, editors. POCT for determination of basic pharmacogenetic profile for individualization of antiplatelet therapy: pilot study. EUROPEAN HEART JOURNAL; 2014: OXFORD UNIV PRESS GREAT CLARENDON ST, OXFORD OX2 6DP, ENGLAND.

10. Roberts JD, Wells GA, Le May MR, Labinaz M, Glover C, Froeschl M, et al. Point-of-care genetic testing for personalisation of antiplatelet treatment (RAPID GENE): a prospective, randomised, proof-of-concept trial. The Lancet.

11. So DY, Wells GA, McPherson R, Labinaz M, Le May MR, Glover C, et al. A prospective randomized evaluation of a pharmacogenomic approach to antiplatelet therapy among patients with ST-elevation myocardial infarction: the RAPID STEMI study. Pharmacogenomics journal. 2016;16(1).

12. Voicu V, Diehm N, Moarof I, Parejo S, Badique F, Burden A, et al. Personalized Antiplatelet Therapy based on Point-of-Care CYP2C19 Pharmacogenetics plus Multidimensional Treatment Decisions in a Cohort of 167 Patients. Swiss Medical Weekly. 2024;154:132S.

13. Voicu V, Diehm N, Moarof I, Parejo S, Badique F, Burden A, et al. Antiplatelet therapy guided by CYP2C19 point-of-care pharmacogenetics plus multidimensional treatment decisions. Pharmacogenomics. 2024;25(1):5-19.

14. Wirth F, Zahra G, Xuereb RG, Barbara C, Fenech A, Azzopardi LM. Comparison of a rapid point-of-care and two laboratory-based CYP2C19*2 genotyping assays for personalisation of antiplatelet therapy. International journal of clinical pharmacy. 2016;38(2).

15. Wirth F, Zahra G, Xuereb RG, Barbara C, Fenech A, Azzopardi LM. Comparison between a point-of-care and a laboratory-based CYP2C19* 2 genotyping assay for pharmacist-led personalisation of antiplatelet therapy. 2015.

16. Al-Rubaish AM, Al-Muhanna FA, Alshehri AM, Alsulaiman AA, Alabdulali MM, Alkhamis F, et al. Prevalence of CYP2C19*2 carriers in Saudi ischemic stroke patients and the suitability of using genotyping to guide antiplatelet therapy in a university hospital setup. Drug metabolism and personalized therapy. 2021;37(1).

17. Azzahhafi J, Broek WWAvd, Chan Pin Yin DRPP, Harmsze AM, van Schaik RHN, Ten Berg JM. The Clinical Implementation of CYP2C19 Genotyping in Patients with an Acute Coronary Syndrome: Insights From the FORCE-ACS Registry. Journal of cardiovascular pharmacology and therapeutics. 2023;28:10742484231210704.

18. Azzahhafi J, van den Broek W, Chan Pin Yin D, Van Schaik R, Berg JT. TCT-381 The Clinical implementation of CYP2C19 Genotyping in Patients With an Acute Coronary Syndrome: Insights From the FORCE-ACS Registry. Journal of the American College of Cardiology. 2023;82(17):B152.

19. Bergmeijer TO, Vos GJ, Claassens DM, Janssen PW, Harms R, der Heide Rv, et al. Feasibility and implementation of CYP2C19 genotyping in patients using antiplatelet therapy. Pharmacogenomics. 2018;19(7).

20. Cavallari LH, Franchi F, Rollini F, Been L, Rivas A, Agarwal M, et al. Clinical implementation of rapid CYP2C19 genotyping to guide antiplatelet therapy after percutaneous coronary intervention. Journal of translational medicine. 2018;16(1).

21. Davis BH, DeFrank G, Limdi NA, Harada S. Validation of the Spartan RXCYP2C19 Genotyping Assay Utilizing Blood Samples. Clinical and translational science. 2020;13(2).

22. Franchi F, Rollini F, Rivas J, Rivas A, Agarwal M, Briceno M, et al. Prasugrel Versus Ticagrelor in Patients With CYP2C19 Loss-of-Function Genotypes: results of a Randomized Pharmacodynamic Study in a Feasibility Investigation of Rapid Genetic Testing. JACC: basic to translational science. 2020;5(5).

23. Gurbel PA, Bliden K, Sherwood M, Taheri H, Tehrani B, Akbari M, et al. Development of a routine bedside CYP2C19 genotype assessment program for antiplatelet therapy guidance in a community hospital catheterization laboratory. Journal of thrombosis and thrombolysis. 2024;57(4):566-75.

24. Gurbel PA, Bell R, Bliden K, Yazdani S, Taheri H, Akbari M, et al. Bedside Testing of CYP2C19 Genotype to Guide Antiplatelet Therapy: Implementation in the Catheterization Laboratory. Journal of the American College of Cardiology. 2018;71(11S):A1202-A.

25. Gurbel PA, Bliden K, Sherwood M, Yazdani S, Taheri H, Truesdell A, et al. Point-of-care CYP2C19 genotype guided antiplatelet therapy in cardiac catheterization laboratory and clinical outcomes after PCI. Journal of the American College of Cardiology. 2019;73(9S1):1329-.

26. Levens AD, den Haan MC, Jukema JW, Heringa M, van den Hout WB, Moes DJAR, et al. Feasibility of Community Pharmacist-Initiated and Point-of-Care CYP2C19 Genotype-Guided De-Escalation of Oral P2Y12 Inhibitors. Genes. 2023;14(3).

27. McDermott JH AS, Wright S, Sen D, Miele G, Smith CJ, Payne k , Newman W, editor Development of a Point-of-Care Pharmacogenetic Test for CYP2C19 Allowing Genotype Guided Antiplatelet Prescribing to Prevent Recurrent Strokes2020.

28. Koltowski L, Tomaniak M, Aradi D, Huczek Z, Filipiak KJ, Kochman J, et al. Optimal aNtiplatelet pharmacotherapy guided by bedSIDE genetic or functional TESTing in elective PCI patients: A pilot study: ONSIDE TEST pilot. Cardiology journal. 2017;24(3).

29. Zhou Y, Armstead AR, Coshatt GM, Limdi NA, Harada S. Comparison of Two Point-of-Care CYP2C19 Genotyping Assays for Genotype-Guided Antiplatelet Therapy. Annals of clinical and laboratory science. 2017;47(6).

30. Zhou Y, Armstead A, Coshatt G, Brott B, Sankaranarayanan A, Limdi N, et al., editors. Rapid CYP2C19 Genotype Testing: Comparison between Spartan RX CYP2C19 and Verigene CYP2C19. JOURNAL OF MOLECULAR DIAGNOSTICS; 2015: ELSEVIER SCIENCE INC 360 PARK AVE SOUTH, NEW YORK, NY 10010-1710 USA.

31. McDermott J, Ainsworth S, Wright S, Sen D, Miele G, Smith C, et al., editors. Development of a Point of Care Test for CYP2C19Allowing Genotype Guided Antiplatelet Prescribing to Prevent Recurrent Ischaemic Strokes. EUROPEAN JOURNAL OF HUMAN GENETICS; 2020: SPRINGERNATURE CAMPUS, 4 CRINAN ST, LONDON, N1 9XW, ENGLAND.
